# MicroRNA‐92b in the skeletal muscle regulates exercise capacity via modulation of glucose metabolism

**DOI:** 10.1002/jcsm.13377

**Published:** 2023-11-20

**Authors:** Shu Yang, Guangyan Yang, Xinyu Wang, Lixing Li, Yanchun Li, Jiaqing Xiang, Lin Kang, Zhen Liang

**Affiliations:** ^1^ Department of Geriatrics, Shenzhen People's Hospital (The Second Clinical Medical College Jinan University Guangzhou China; ^2^ The First Affiliated Hospital Southern University of Science and Technology Shenzhen China; ^3^ Guangdong Provincial Clinical Research Center for Geriatrics, Shenzhen Clinical Research Centre for Geriatrics Shenzhen People's Hospital Shenzhen China; ^4^ The Biobank of National Innovation Center for Advanced Medical Devices Shenzhen People's Hospital Shenzhen China

**Keywords:** Exercise capacity, Glycogen synthesis, Lactate extrusion, miR‐92b, Skeletal muscle

## Abstract

**Background:**

Exercise mimetics is a proposed class of therapeutics that specifically mimics or enhances the therapeutic effects of exercise. Muscle glycogen and lactate extrusion are critical for physical performance. The mechanism by which glycogen and lactate metabolism are manipulated during exercise remains unclear. This study aimed to assess the effect of miR‐92b on the upregulation of exercise training‐induced physical performance.

**Methods:**

Adeno‐associated virus (AAV)‐mediated skeletal muscle miR‐92b overexpression in C57BLKS/J mice, and global knockout of miR‐92b mice were used to explore the function of miR‐92b in glycogen and lactate metabolism in skeletal muscle. AAV‐mediated UGP2 or MCT4 knockdown in WT or miR‐92 knockout mice was used to confirm whether miR‐92b regulates glycogen and lactate metabolism in skeletal muscle through UGP2 and MCT4. Body weight, muscle weight, grip strength, running time and distance to exhaustion, and muscle histology were assessed. The expression levels of muscle mass‐related and function‐related proteins were analysed by immunoblotting or immunostaining.

**Results:**

Global knockout of miR‐92b resulted in normal body weight and insulin sensitivity, but higher glycogen content before exercise exhaustion (0.8538 ± 0.0417 vs. 1.043 ± 0.040, **P = 0.0087), lower lactate levels after exercise exhaustion (4.133 ± 0.2589 vs. 3.207 ± 0.2511, *P = 0.0279), and better exercise capacity (running distance to exhaustion, 3616 ± 86.71 vs. 4231 ± 90.29, ***P = 0.0006; running time to exhaustion, 186.8 ± 8.027 vs. 220.8 ± 3.156, **P = 0.0028), as compared with those observed in the control mice. Mice skeletal muscle overexpressing miR‐92b (both miR‐92b‐3p and miR‐92b‐5p) displayed lower glycogen content before exercise exhaustion (0.6318 ± 0.0231 vs. 0.535 ± 0.0194, **P = 0.0094), and higher lactate accumulation after exercise exhaustion (4.5 ± 0.2394 vs. 5.467 ± 0.1892, *P = 0.01), and poorer exercise capacity (running distance to exhaustion, 4005 ± 81.65 vs. 3228 ± 149.8, ***P<0.0001; running time to exhaustion, 225.5 ± 7.689 vs. 163 ± 6.476, **P = 0.001). Mechanistic analysis revealed that miR‐92b‐3p targets UDP‐glucose pyrophosphorylase 2 (UGP2) expression to inhibit glycogen synthesis, while miR‐92b‐5p represses lactate extrusion by directly target monocarboxylate transporter 4 (MCT4). Knockdown of UGP2 and MCT4 reversed the effects observed in the absence of miR‐92b in vivo.

**Conclusions:**

This study revealed regulatory pathways, including miR‐92b‐3p/UGP2/glycogen synthesis and miR‐92b‐5p/MCT4/lactate extrusion, which could be targeted to control exercise capacity.

## Introduction

Increased physical activity has been demonstrated to have positive effects in preventing and ameliorating a wide range of diseases, including brain disorders, such as Alzheimer's disease, dementia, cancer, diabetes and cardiovascular disease.^1^, ^2^ Exercise mimetics can be used to promote skeletal muscle protein synthesis and delay skeletal muscle decay, by simulating biochemical and functional responses to regular exercise.^3^ In mice, it has been shown that oral administration of AICAR, a drug targeting the AMPK‐peroxisome proliferator‐activated receptor‐delta pathway, enhanced training adaptations and even improved the degree of endurance in the absence of exercise.^3^ Similarly, many substances can lead to increased skeletal muscle mass and enhanced performance, including REV‐ERB alpha agonists, sirtuin 1, and peroxisome proliferator‐activated receptors.^4^

Skeletal muscle glycogen is critical for exercise capacity, as it is the main energy fuel used in muscle contraction.^5^ A strong association has been shown between muscle glycogen depletion, impaired muscle performance, and fatigue development during exercise.^6^, ^7^ This association is also supported by findings in patients with glycogen storage disease, which is characterized by complete elimination of muscle gys1, leading to muscle weakness, pain, cramps and poor exercise performance, with a low maximal workload, and death due to cardiac events in childhood.^8^, ^9^ Notably, endurance exercise training increases the rate and magnitude of muscle glycogen supercompensation in rats^10^; however, the molecular mechanism by which exercise training promotes an increase in glycogen synthesis in the skeletal muscle remains unclear. In the process of gradually increasing exercise load, the concentration of blood lactic acid increases with a gradual increase in exercise load.^11^ Upon exercising hard, more lactic acid is produced, which blocks the ability of the muscles and blood to supply oxygen.^11^ Hence, a higher capacity for lactate exchange and removal from skeletal muscle is associated with high exercise performance.^12^ However, the molecular mechanism by which exercise affects lactate threshold in skeletal muscles remains unclear.

Small non‐coding RNAs serve a wide range of regulatory roles and have increasingly been recognized as therapeutic targets.^13^ One of the best‐studied classes of small non‐coding RNAs are microRNAs (miRNAs), which can act as ‘master regulators’ of gene expression.^14^ It had been reported that miR‐92b is involved in the regulation of Akt signalling, both in vivo and in vitro. PTEN, a negative regulator of Akt signalling, is directly associated with miR‐92b.^15^, ^16^ Another study showed that miR‐92b‐3p overexpression suppressed Gabra3 expression, which led to the inactivation of important oncogenic pathways, including the Akt/mTOR and JNK pathways.^17^ In addition, exercise has been shown to modulate the expression of various small non‐coding RNAs, including miRNAs, and aerobic exercise training remodels miRNA expression in the skeletal muscle.^18^ Several miRNAs are altered in response to acute aerobic exercise,^19^ indicating the potential of miRNA‐dependent regulation of skeletal muscle exercise adaptation. However, the direct role of specific miRNAs in the regulation of gene expression and metabolism in the skeletal muscle, in response to exercise training, remains unknown.

## Methods

### MicroRNA‐92b knockout mice

Male miR‐92b^−/−^ mice with a complete deletion of exons were purchased from the Animal Center of Nanjing University (Nanjing, Jiangsu, China). Eight‐ and 10‐week‐old male miR‐92b^−/−^ mice were used in this study. Age‐matched C57BL/6 mice were used as the WT controls. Mice were maintained in a temperature‐controlled (25°C) facility with a 12 h/12 h light/dark cycle and provided free access to food and water, except when fasting blood specimens were obtained.

### Diabetic mice

MiR‐92b^−/−^ and C57BL/6J mice (8 ± 0.5 weeks old and 24 ± 1 g) were randomly divided into two groups consisting of *n* = 6 animals each. After being fed HFD (D12492; Research Diets, New Brunswick, NJ, USA) for 4 weeks, mice were intraperitoneally administered 50 mg/kg body weight STZ for 5 days. After STZ administration, the diabetic mice were maintained on HFD diet for 10 weeks.

### Systemic injection of AAV9‐hsa‐miR‐92b into C57BL/6J mice

AAV9‐hsa‐miR‐92b (GeneChem Company, Shanghai, China) or AAV9‐hsa‐*gfp* (1 × 10^12^ vg/mL in 200 μL saline [0.15 mol/L NaCl]; GeneChem Company) was injected into the tail vein of C57BL/6J mice, to induce miR‐92b overexpression (*n* = 6 per group).

### UGP2 or MCT4 knockdown in the skeletal muscle of M92KO mice

To induce knockdown of UGP2 or MCT4 in the skeletal muscle of WT or M92KO mice, AAV9‐hsa‐short hairpin (sh) *Ugp2* or AAV9‐hsa‐sh*Mct4* (GeneChem Company) (1 × 10^12^ vg/mL in 50 μL saline [0.15 mol/L NaCl]) was injected into the tail vein of WT or M92KO mice, as indicated (*n* = 6 per group). AAV9‐hsa‐null‐transfected WT or M92KO mice were used as the control group.

### Data analysis

All the data were generated from at least three independent experiments. No data, sample cells or mice were excluded from statistical analysis. Data are expressed as mean ± SD of each group. Based on our previous studies and/or preliminary experiments, we calculated the group size required for in vivo and in vitro studies. The density of images was quantified by an individual who was blinded to the treatment, using ImageJ software (version 1.51; NIH). The density of each target band was normalized to β‐actin in the corresponding sample to reduce variance. The qPCR data and western blot quantifications were expressed as fold of the control group's mean value, which was defined as 1 plus its own SD. A two‐tailed unpaired Student's *t* test was used for comparison between two groups. Comparisons between more than two experimental groups were conducted by one‐way ANOVA, followed by be adjusted for False Discovery Rate. All analyses were carried out using GraphPad Prism 7.04 (www.graphpad.com/). *P* < 0.05 was considered statistically significant.

## Results

### miR‐92b expression is regulated by exercise in the skeletal muscle

**Figure 1:**
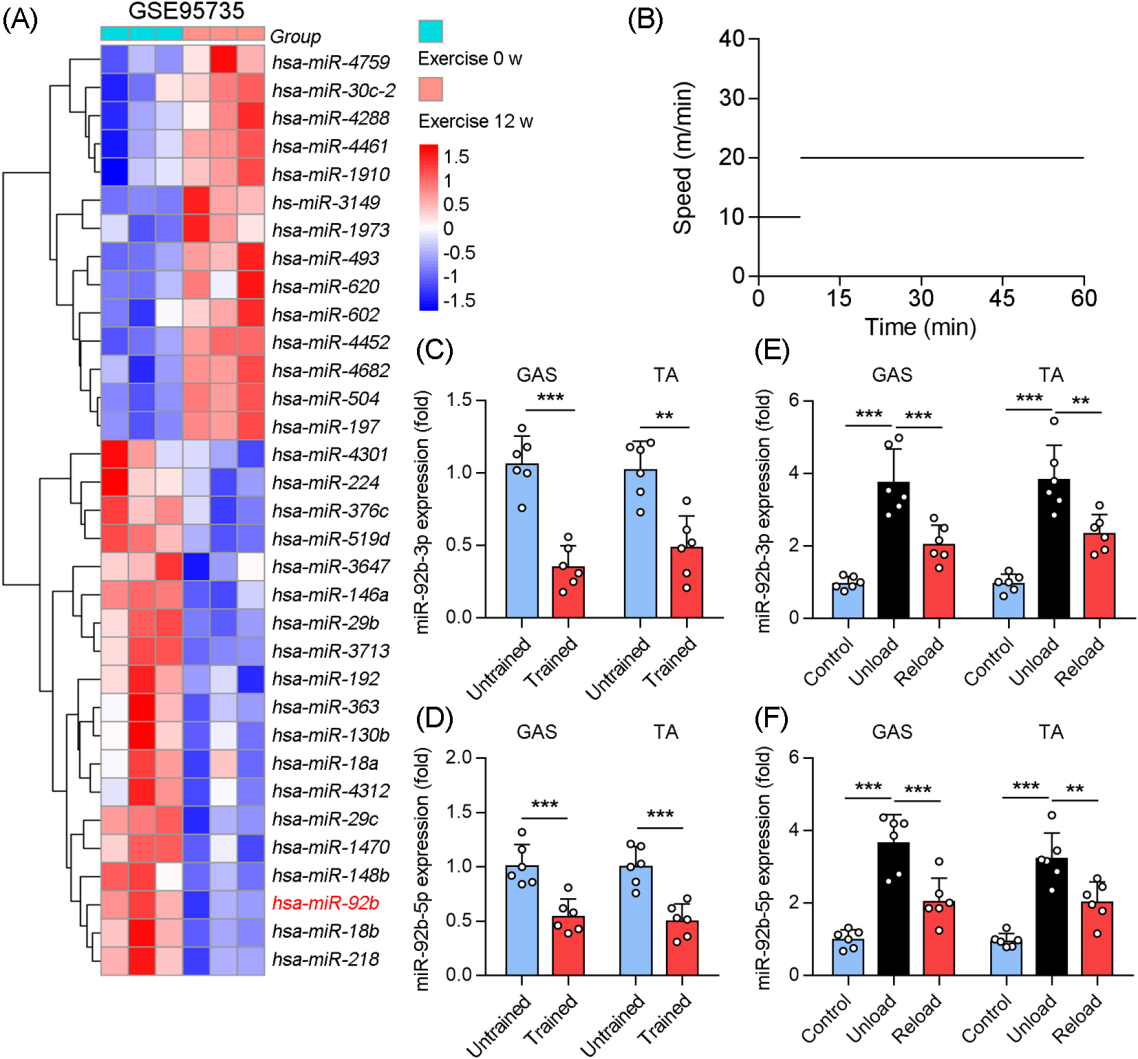
Skeletal muscle miR‐92b was downregulated by long‐term exercise training. (A) Reanalyzed the database obtained from GEO database (GSE95735) on human specimens from vastus lateralis before and after 12 weeks of endurance exercise (*n* = 3). (B) The pattern of treadmill speed for training (45 min daily for 4 weeks). (C) The qPCR was used to determine miR‐92b‐3p levels in the Gas and TA muscle from untrained and trained mice, *n* = 6. (D) qPCR was used to determine miR‐92b‐5p expression in Gas and TA muscle from untrained and trained mice, *n* = 6. (E) The qPCR was used to determine miR‐92b‐3p levels in the Gas and TA from WT mice in control (nonsuspension), unload (suspension for 7 days), and reload (7 days reload after 7 days suspension), *n* = 6. (F) qPCR was used to determine miR‐92b‐5p expression in Gas and TA muscle from WT mice in control (nonsuspension), unload (suspension for 7 days), and reload (7 days reload after 7 days suspension), *n* = 6. All results are expressed as means ± SD. *P < 0.05, **P < 0.01, ***P < 0.001, by unpaired Student's *t* test.

We first identified that miR‐92b expression in the vastus lateralis decreased after 12 weeks of endurance exercise, as compared with that in the no exercise control group (Figure 1A; GSE95735). Furthermore, when we compared the miR‐92b‐3p expression in the skeletal muscle between mice subjected to long‐term training exercise (Figure 1B) and those subjected to no exercise, its expression in the gastrocnemius (Gas) and tibialis anterior (TA) muscles was significantly lower in the exercise mice (Figure 1C). In agreement with this, the long‐term training exercise mice displayed a decrease in miR‐92b‐5p expression in the Gas and TA (Figure 1D). Hindlimb suspension (HLS) of rodents by the tail is a well‐established approach to create a ground‐based model of microgravity and musculoskeletal disuse that mimics many of the physiological changes associated with space flight, prolonged bed rest, as well as extreme non‐exercising.^20^, ^21^ C57BL/6J mice were randomly divided into three groups [control (non‐suspension), unload (7 days of suspension), reload (reloaded for 7 days, after 7 days of HLS)]. As compared with those in the control group, the weights of the Gas and TA muscles were significantly lower in the unload group and recovered in the reload group (Figure S1A, B). MiR‐92b‐3p and miR‐92b‐5p mRNA levels were higher in the Gas and TA muscles from the HLS mice, but lower after 7 days of reloading, although not back to normal (Figure 1E,F). In addition, miR‐92b‐3p and miR‐92b‐5p levels did not change significantly in the fat tissue and heart after exercise (data not shown), indicating that the increased expression of miR‐92b after exercise is not a systemic effect. These data suggested that miR‐92b‐3p and miR‐92b‐5p in the skeletal muscle are correlated with physical activity.

### MiR‐92b knockout (M92KO) mice show increased exercise capacity

**Figure 2:**
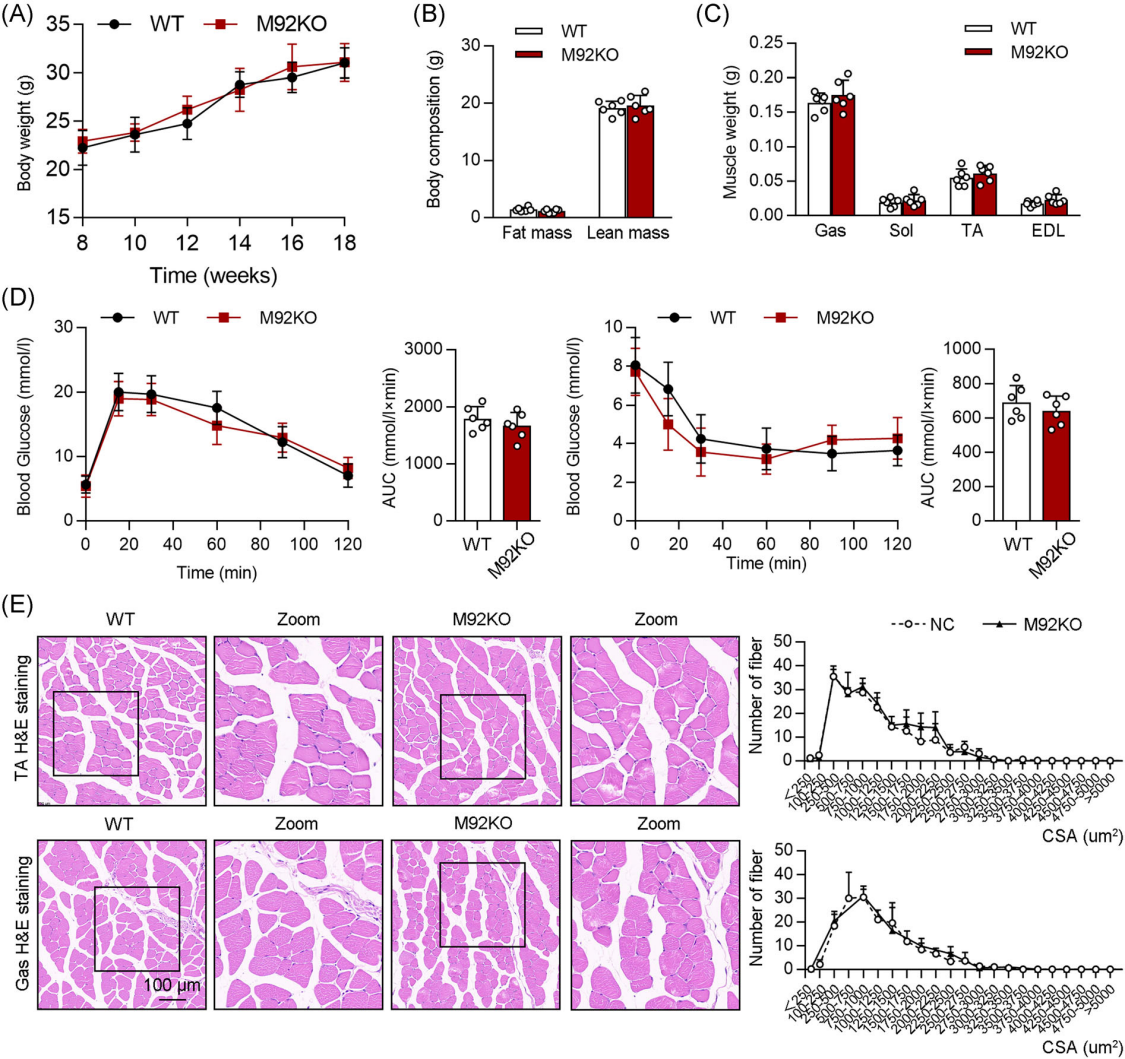
Deficiency of miR‐92b shows normal systemic metabolism. (A) Body weight (BW) changes over time of WT and miR‐92b knockout (M92KO) mice (*n* = 6). (B) Body mass measured by MRI in 18‐week‐old mice (*n* = 6). (C) Weights of four muscle types, Gas, Sol, TA, and digitorum longus (EDL) from WT or M92KO mice (*n* = 6). (D) Blood glucose during GTT (left panel) and ITT (right panel) (*n* = 6). (E) Histology of TA (the upper panel) and Gas (the bottom panel) muscles, as determined by H&E staining in both the WT or M92KO mice, and quantitative analysis of findings (*n* = 6). All results are expressed as means ± SD. *P < 0.05, **P < 0.01, ***P < 0.001, by unpaired Student's *t* test.

We further evaluated the role of miR‐92b in the skeletal muscle using M92KO mice. Analysis of the levels of miR‐92b‐3p and miR‐92b‐5p in the Gas verified the knockout of miR‐92b in the skeletal muscles of the mice (Figure S2A), with no influence on the heart weight/tibia length ratio (Figure S2B) or daily food intake (Figure S2C). On a regular chow diet, we also found no evident difference in body weight and body composition between the wild‐type (WT) and M92KO mice (Figure 2A,B). In agreement with this, miR‐92b^−/−^ mice displayed similar muscle weights (Gas; soleus, Sol; TA; extensor digitorum longus, EDL), as compared with the WT mice (Figure 2C). Skeletal muscle is a major component of glucose metabolism. M92KO did not significantly influence the fasting blood glucose (FBG) and the refed blood glucose (Figure 2D). The mice also showed normal results in the insulin tolerance test (ITT) and glucose tolerance test (GTT) (Figure 2D). We also examined the skeletal muscle using microscopy to see whether the absence of miR‐92b had any gross morphological consequences. Examination of skeletal muscle sections from the M92KO mice using haematoxylin and eosin (H&E) staining did not reveal any abnormalities (Figure 2E).

**Figure 3:**
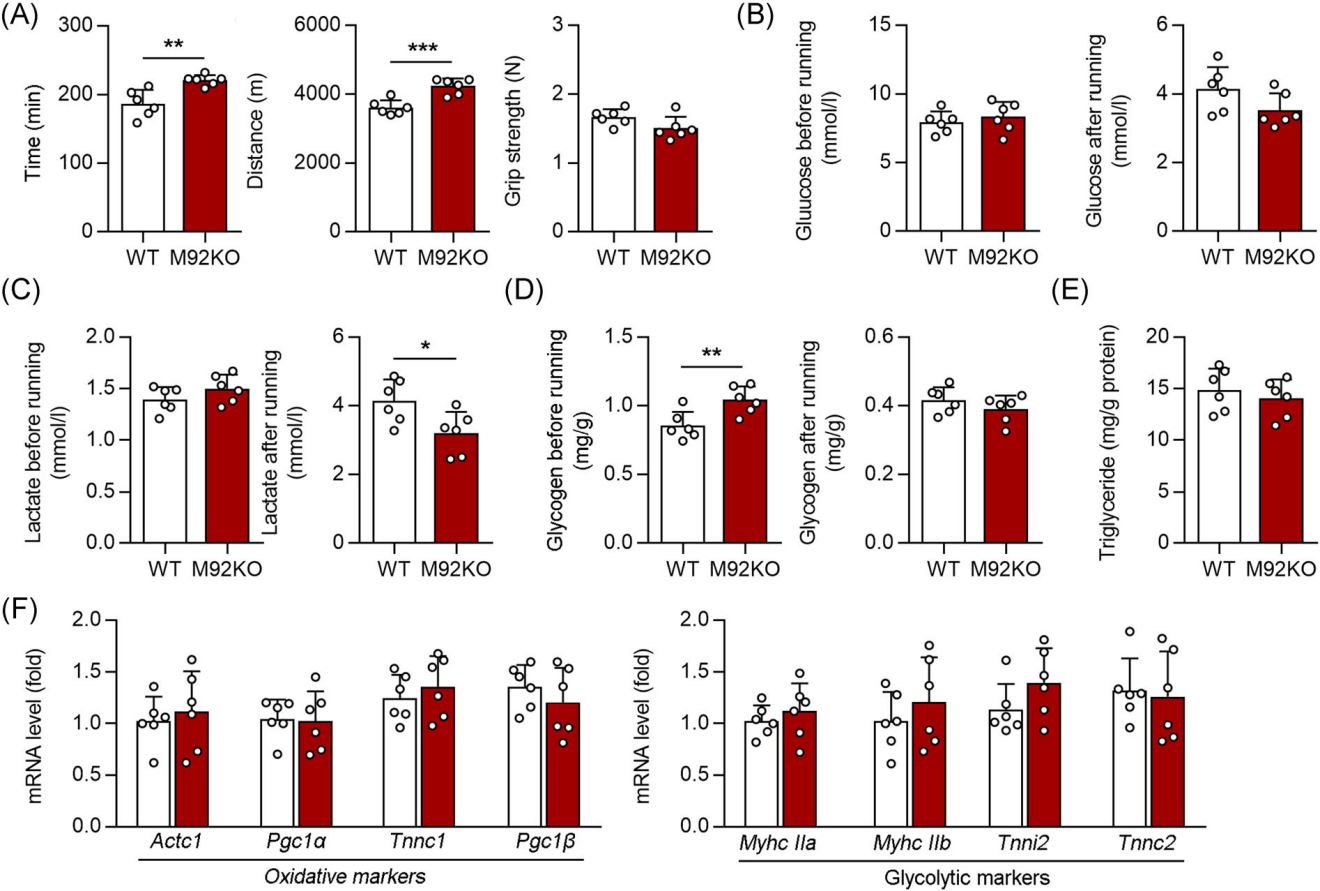
Loss of miR‐92b increases the exercise capacity. (A) The running time (left) and distance (middle) to exhaustion, and forelimb grip strength (right) of WT and M92KO mice, *n* = 6. (B) The glucose level of WT and M92KO mice before and after running exhaustion (*n* = 6). (C) The lactate level of WT and M92KO mice before and after running exhaustion (*n* = 6). (D) The glycogen level of WT and M92KO mice before and after running exhaustion (*n* = 6). (E) The triglyceride level of WT and M92KO mice before running exhaustion. (F) qPCR analysis was used to detect mRNA levels of *Actc1*, *Pgc1α* (also known as Ppargc1α), *Tnnc1*, and *Pgc1β* (also known as Ppargc1β), and *Myhc IIa* (also known as Myh2), *Myhc IIb* (also known as Myh4), *Tnni2* and *Tnnc2* in Gas muscle (*n* = 6) from WT and M92KO mice. All results are expressed as means ± SD. *P < 0.05, **P < 0.01, ***P < 0.001, by unpaired Student's *t* test.

Next, we determined whether knocking out miR‐92b affects exercise capacity, by subjecting M92KO and WT mice to a high‐intensity treadmill exercise regimen. Intriguingly, we found that knockout of miR‐92b enhanced the exhaustive running time and maximum running distance, but not the grip strength of the forelimbs of the mice (Figure 3A). We next measured blood glucose levels before and after running, to confirm that all mice ran to their limit of fatigue, and to test whether differences in blood glucose levels might have affected exercise capacity. Blood glucose levels declined with exercise in both the groups, with no significant difference in pre‐ or post‐exercise levels between the groups (Figure 3B). Lactate formation causes acidosis and muscular fatigue.^22^ The lactate levels in the Gas from WT and M92KO mice were evidently elevated after exercise, while the M92KO mice displayed a lower level of lactate in the Gas than WT mice (Figure 3C), indicating that the increased exercise capacity of M92KO mice may result from decreased lactate content in the skeletal muscle after exercise. As glycogen depletion can contribute to exercise fatigue (‘glycogen shunt’ hypothesis^7^), we assessed baseline glycogen levels in the muscles of both the groups, and found that the miR‐92b^−/−^ mice had more glycogen content in the skeletal muscle than the WT mice, before exercise, but not after exercise (Figure 3D), indicating that another reason for the increased exercise capacity of M92KO mice could be from the increased glycogen content before exercise. In addition, there was no difference in the skeletal muscle triglyceride content between the WT and miR‐92b^−/−^ mice (Figure 3E). We determined the mRNA levels of oxidative and glycolytic myofiber markers in the Gas of WT and M92KO mice, but found no differences in the muscle‐fibre‐type genes (Figure 3F).

### The skeletal muscle in the miR‐92b‐overexpressing (M92OE) mice shows reduced exercise capacity

**Figure 4:**
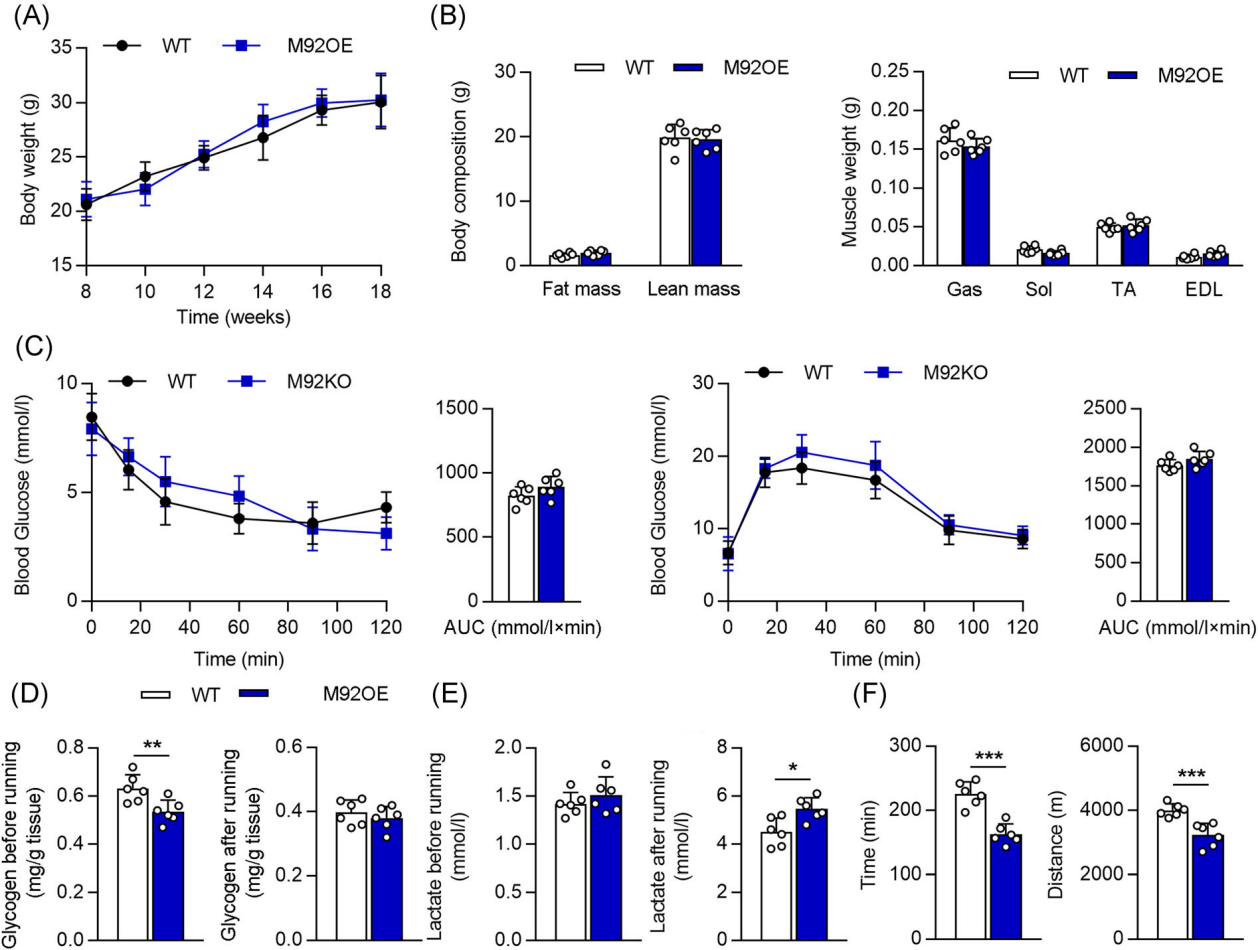
MiR‐92b overexpression in skeletal muscle reduced the exercise capacity. (A) Body weight (BW) changes over time of WT and miR‐92b overexpression (M92OE) mice (*n* = 6). (B) Body mass measured by MRI,amd weights of four muscle types, Gas, Sol, extensor, TA, and EDL from WT or M92OE mice (*n* = 6). (C) Blood glucose during ITT (left panel) and GTT (right panel) (*n* = 6). (D) The glycogen level of WT and M92OE mice before and after running exhaustion (*n* = 6). (E) The lactate level of WT and M92OE mice before and after running exhaustion (*n* = 6). (F) The running time (left) and distance (right) to exhaustion of WT and M92OE mice (*n* = 6). All results are expressed as means ± SD. *P < 0.05, **P < 0.01, ***P < 0.001, by unpaired Student's *t* test.

Systemic injection of AAV9 in vivo can efficiently target skeletal muscles and peripheral organs.^23^ Moreover, it had been reported that the Cre recombinase is expressed in the skeletal muscles (gastrocnemius and quadriceps) of HSA‐Cre (muscle‐specific Acta1, encoding HSA) mice treated with tamoxifen, but not in the liver or heart.^24^ Consistently, a study showed that AAV9‐hsa‐miR‐193b transfection had no obviously the expression of miR‐193b in heart of mice.^23^ Hence, the muscle‐specific HSA promoter was used to drive target gene expression in the muscles. C57BL/6J mice were injected with AAV9‐Ctrl (control group) or AAV9‐hsa‐miR92b (M92OE) via the tail vein, following which the muscles were harvested 10 weeks after injection. Compared with that in the control mice, miR‐92b‐3p and miR‐92b‐5p expression was significantly increased in the Gas of M92OE mice (Figure S3A), but not in the other organs, such as the liver, heart, forebrain, cerebellum, fat, and kidney (data not shown). There were no significant differences in body weight, body composition and muscle weight (Gas, Sol, TA, and EDL) between the control and M92OE mice (Figure 4A,B). Furthermore, M92OE mice displayed similar GTT and ITT results as the control mice fed a chow diet (Figure 4C). In addition, there were similar levels of FBG, fasting blood insulin, and refed blood glucose in the control and M92OE mice (Figure S3B). Not surprisingly, M92OE mice displayed significantly lower glycogen content in the Gas before exercise than control mice, but similar contents after exercise (Figure 4D). Further analysis of the lactate content in the Gas revealed that miR‐92b overexpression significantly increased the lactate level in the M92OE mice, as compared with that in the control mice, after exercise, but not before exercise (Figure 4E). Finally, compared with the control mice, the M92OE mice showed a reduction in exercise capacity, as evidenced by a reduction in exhaustive running time and maximum running distance (Figure 4F).

### Knocking out miR‐92b improved insulin resistance and muscle loss in diabetic mice

**Figure 5:**
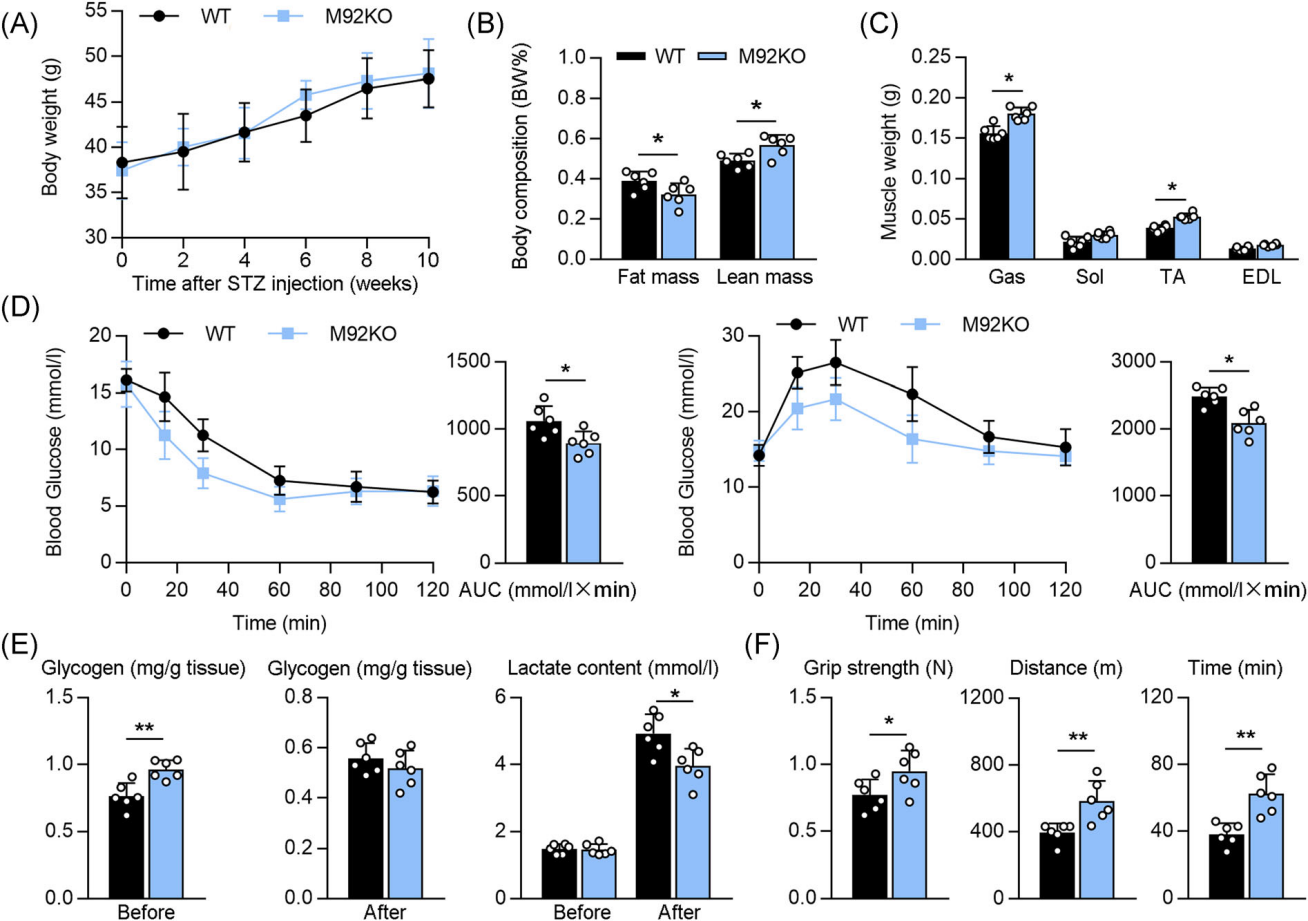
Absent of miR‐92b improved insulin resistance and enhanced the exercise capacity in diabetic mice. (A) Body weight (BW) changes over time of WT or M92KO mice made in diabetes model (*n* = 6). (B) Body mass measured by MRI in diabetic mice (*n* = 6). (C) Weights of four muscle types, Gas, Sol, extensor, TA, and EDL from diabetic mice (*n* = 6). (D) Blood glucose during ITT (left panel) and GTT (right panel) (*n* = 6). (E) The glycogen level (left and middle) and lactate level (right) of WT and M92KO mice before and after running exhaustion (*n* = 6). (F) The forelimb grip strength (left), and the running time (right) and distance (middle) to exhaustion of WT and M92KO mice (*n* = 6). All results are expressed as means ± SD. *P < 0.05, **P < 0.01, ***P < 0.001, by unpaired Student's *t* test.

Muscle glycogen synthesis comprises a principal pathway of glucose storage. Impairment of muscle glucose uptake and glycogen synthesis are major contributors to insulin resistance and type 2 diabetes mellitus.^25^ Hence, M92KO mice were fed a high‐fat diet (HFD) and treated with streptozotocin (STZ) to establish the diabetic model (Figure S4A, B). M92KO did not affect the ratio of heart weight and tibia length, daily food intake or body weight in the HFD diet (Figure 5A and S4C). However, M92KO mice had a higher ratio of lean mass to body weight and a lower ratio of fat mass to body weight than WT mice (Figure 5B). In agreement with this, the Gas and TA muscle weights were significantly higher in the M92KO mice than in the WT mice (Figure 5C). Next, we found that the glucose level was reduced in the ITTs and GTTs, resulting in a decrease in the area under the curve for glucose (Figure 5D). In addition, compared with that in the control mice, we found that M92KO reduced insulin resistance in the diabetic mice, as shown by decreased fasting and refed glucose levels, as well as, fasting insulin levels in the serum (Figure S4D). Consistent with our findings in Figure 3D, M92KO mice displayed significantly higher glycogen content in the Gas than WT mice, before exercise, but not after exercise (Figure 5E). Exercise training increased the lactate content in the Gas of both WT and M92KO mice, and M92KO mice had a significantly lower level of lactate after exercise than WT mice (Figure 5E). Furthermore, our results indicated that M92KO strengthened the grip strength of the forelimb of mice and increased exhaustive running time and maximum running distance, as compared with those observed in the WT mice (Figure 5F). Muscle mass is controlled by the balance between protein synthesis and degradation.^26^ M92KO enhanced the phosphorylation of Akt, mTOR, and S6K in the Gas, as compared with that observed in the WT mice, while it reduced the expression of Atrogin‐1 and MuRF1 (muscle‐specific ubiquitin ligases, Figure S4E, F). Collectively, these data suggested that M92KO inhibited muscle loss and weakness, as well as, fortified AKT/mTOR/S6K activation in the muscles of the diabetic mice.

### MiR‐92b‐3p potentially regulates glycogen synthesis in the skeletal muscle through UGP2

**Figure 6:**
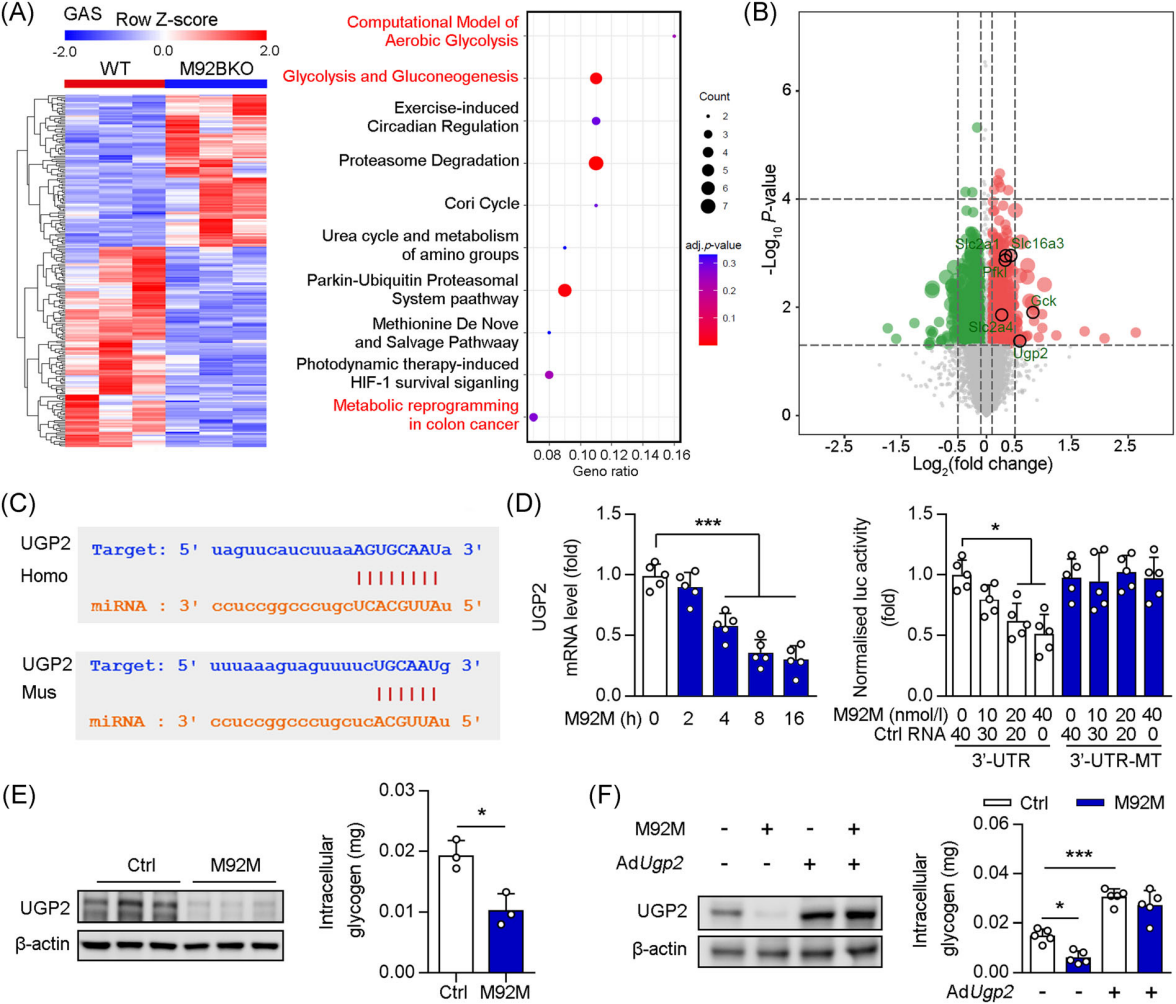
UGP2 was a directly target of miR‐92b‐3p in skeletal muscle. (A, B) Heat map of differentially expressed genes in Gas of WT and M92KO mice (*n* = 3), and the Wiki Pathway Analysis of RNA‐seq on GAS, and volcano plot of differentially expressed genes in GAS of WT and M92KO mice (*P* < 0.05). (C) Graphical representation of the conserved miR‐92b‐3p binding motifs within the 3′‐UTR of UGP2. Complementary sequences to the seed regions of miR‐92b‐3p within the 3′‐UTRs are conserved between human (Homo) and mouse (Mus) sequences. (D) C2C12 cells were treated with miR‐92b‐3p mimic or control (Ctrl) RNA and qPCR analysis was used to determine the mRNA level of UGP2 (*n* = 5) and Luciferase (luc) activity of the reporter constructs containing either wild‐type ormutated (MT) 3′‐UTR of murine UGP2 after treatment of C2C12 cells with miR‐92b‐3p mimic (M92M) or Ctrl RNA (*n* = 5). (E) C2C12 cells treated with M92M or Ctrl RNA, the protein level of UGP2 and β‐actin were determined by western blot and the intracellular glycogen was detected by commercial kit (*n* = 3). (F) C2C12 cells treated with M92M and/or Ad*Ugp2*, the protein level of UGP2 and β‐actin were determined by western blot (*n* = 3), and the intracellular glycogen was detected by commercial kit (*n* = 3). All results are expressed as means ± SD. *P < 0.05, **P < 0.01, ***P < 0.001, by unpaired Student's *t* test (E to I). *P < 0.05, **P < 0.01, ***P < 0.001, by a one‐way ANOVA with a Bonferroni correction test (J, K).

To further investigate the molecular changes induced by miR‐92b knockout or overexpression in the skeletal muscle, we performed RNA‐sequencing (RNA‐seq) of Gas muscle samples from WT and M92KO mice, the results for which indicated 2165 differentially expressed genes (DEGs), with a *p*‐value of <0.05 (Figure 6A). Among these, 1119 were upregulated, while 1046 were downregulated, in the M92KO mice, as compared with the WT mice (Figure 6A). Wiki Pathway Analysis of the data showed that the most significant DEGs were related to the computational model of aerobic glycolysis and other pathways involved in glycolysis and gluconeogenesis (Figure 6A). Among these genes, UGP2 (the catalytic enzyme that converts G1P to UDP‐glucose; in the liver and muscle tissue, UDP‐glucose is a direct precursor of glycogen) was the only gene related to glycogen synthesis (Figure 6B). Using StarBase (http://starbase.sysu.edu.cn/), we identified *UGP2* as a potential target of miR‐92b‐3p (Figure 6C). M92KO mice displayed significantly higher *Ugp2* mRNA and protein levels in the Gas than WT mice (Figure S5A, B). Conversely, M92OE significantly reduced the mRNA and protein levels of *Ugp2* in the Gas muscle (Figure S5A, B). When compared with the control RNA treatment, treatment with the miR‐92b‐3p mimic (M92M) reduced the mRNA level of *Ugp2* in a time‐dependent manner in C2C12 cells (Figure 6D). Next, we used a Dual‐Luciferase® reporter assay to show that the activity of luciferase reporters was markedly reduced upon M92M treatment, in a dose‐dependent manner, as compared that seen upon treatment with control RNA alone, while reporter activity was not affected following treatment with the *Ugp2* 3′‐UTR‐mutant luciferase reporter construct **(**Figure 6D), suggesting that *Ugp2* is the direct target gene of miR‐92b‐3p. Analysis of protein levels in C2C12 cells by means of western blot assay also supported our findings that treatment with the miR‐92b‐3p inhibitor (M92I) upregulated the expression of UGP2, as compared with control RNA treatment (Figure S5C). In contrast, M92M inhibited the UGP2 protein level in C2C12 cells, as compared with that observed upon control RNA treatment (Figure 6E). Furthermore, as compared with control RNA treatment, M92I treatment increased the intracellular glycogen content in C2C12 cells, while M92M treatment decreased the same (Figure 6E). Notably, treatment with *Ugp2* small interfering RNA (siRNA) decreased the intracellular glycogen content in C2C12 cells, as compared with control RNA treatment, while there was no change after M92I treatment (Figure S5D). Inversely, adenovirus‐mediated *Ugp2* overexpression increased the intracellular glycogen content, which was unchanged upon M92M treatment (Figure 6F). Taken together, we demonstrated that miR‐92b‐3p regulates glycogen synthesis, by inhibiting *UGP2* expression, through direct binding to the *UGP2* 3′‐UTR.

### MiR‐92b‐5p potentially regulates extrusion of lactate in the skeletal muscle through MCT4

**Figure 7:**
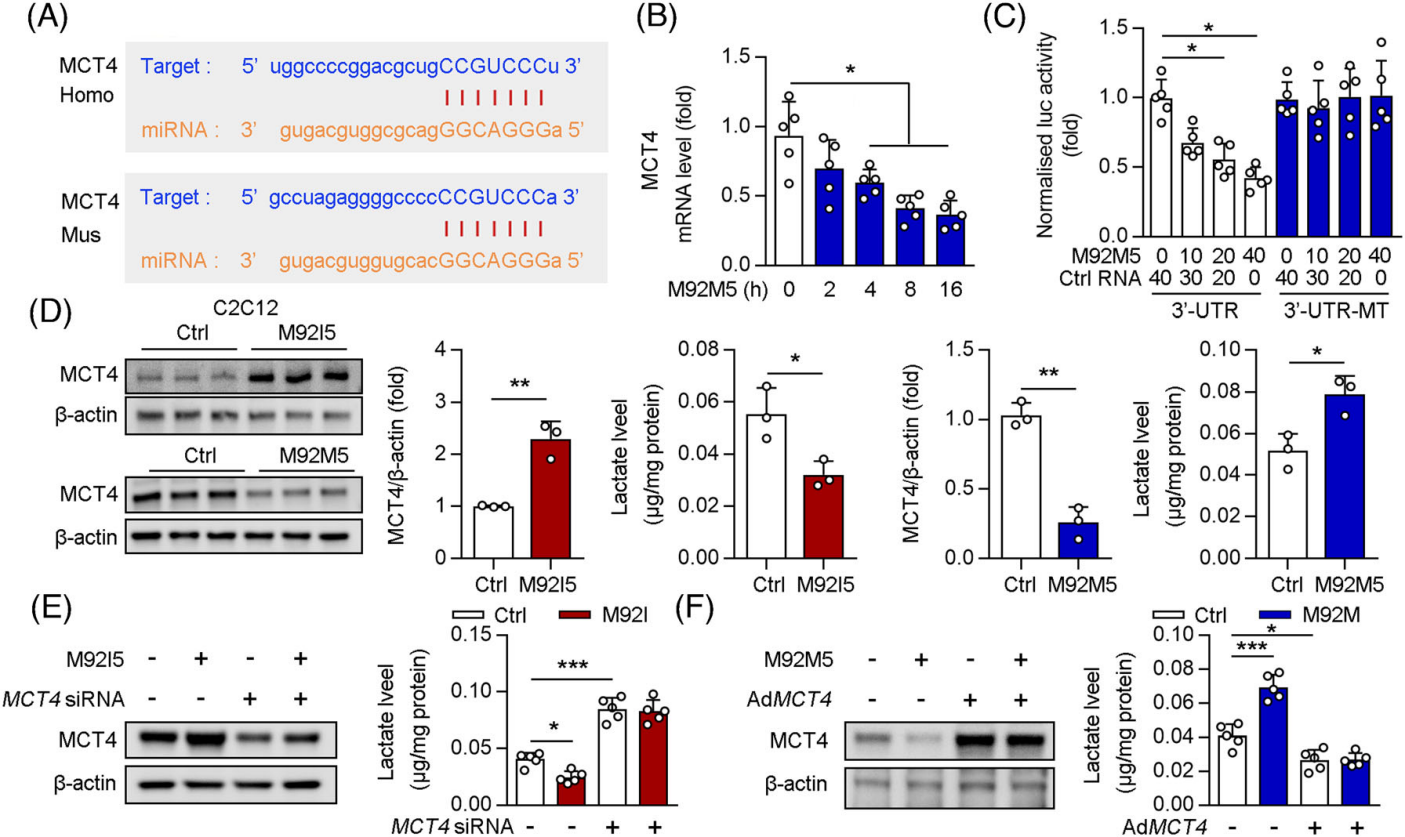
MCT4 was a directly target of miR‐92b‐5p in skeletal muscle. (A) Graphical representation of the conserved miR‐92b‐5p binding motifs within the 3′‐UTR of MCT4. Complementary sequences to the seed regions of miR‐92b‐5p within the 3′‐UTRs are conserved between human (Homo) and mouse (Mus) sequences. (B) C2C12 cells were treated with miR‐92b‐5p mimic or control (Ctrl) RNA and qPCR analysis was used to determine the mRNA level of MCT4 (*n* = 5). (C) Luciferase (luc) activity of the reporter constructs containing either wild‐type ormutated (MT) 3′‐UTR of murine MCT4 after treatment of C2C12 cells with miR‐92b‐5p mimic (M92M5) or Ctrl RNA (*n* = 5). (D) C2C12 cells treated with M92M5 (or miR‐92b‐5p inhibitor, M92I5) or Ctrl RNA under hypoxia, the protein level of MCT4 and β‐actin were determined by western blot and the quantitative result of western blot were shown (*n* = 3), intracellular lactate was detected by commercial kit (*n* = 3). (E) C2C12 cells treated with M92I5 and/or *Mct4* siRNA under hypoxia, the protein level of MCT4 and β‐actin were determined by western blot, and the intracellular lactate was detected by commercial kit (*n* = 3). (F) C2C12 cells treated with M92M5 and/or Ad*Mct4* under hypoxia, the protein level of MCT4 and β‐actin were determined by western blot, and the intracellular lactate was detected by commercial kit (*n* = 3). All results are expressed as means ± SD. *P < 0.05, **P < 0.01, ***P < 0.001, by unpaired Student's *t* test (B to F). *P < 0.05, **P < 0.01, ***P < 0.001, by a one‐way ANOVA with a Bonferroni correction test (G, H).

Among the DEGs related to glucose metabolism, we found that Slc16a3 (MCT4) was significantly upregulated in the Gas muscles from M92KO mice. MCT4 is highly expressed in glycolytic fast‐twitch muscles and is characterized by its role in the extrusion of lactate from muscle cells.^27^, ^28^ Using StarBase, we identified *MCT4* as a potential target of miR‐92b‐5p (Figure 7A). M92KO mice displayed significantly higher *MCT4* mRNA and protein levels in the Gas than WT mice (Figure S5E, F). Conversely, M92OE significantly reduced the mRNA and protein levels of *Mct4* in the Gas muscle (Figure S5E, F). In C2C12 cells, the miR‐92b‐5p mimic (M92M5) decreased the mRNA level of MCT4 in a time‐dependent manner (Figure 7B). To further investigate the interaction between miR‐92b‐5p and MCT4, we constructed two plasmids, MCT4 3′‐UTR and MCT4‐3′‐UTR‐MT, which contained the WT and mutant 3′‐UTR of MCT4, respectively. Each plasmid was co‐transfected with M92M5 into C2C12 cells. Our results showed that M92M5 inhibited the luciferase activity in a dose‐dependent manner (Figure 7C). However, M92M5 did not regulate the luciferase activity in C2C12 cells transfected with the plasmid expressing MCT4‐3′‐UTR‐MT (Figure 7C). In vitro, as compared with treatment with control RNA, M92I5 treatment increased the MCT4 protein level in C2C12 cells, whereas M92M5 treatment reduced the same (Figure 7D). Consistent with this, a reduction in intracellular lactate content was observed upon M92I5 treatment, while an increase in the same was observed upon M92M5 treatment (Figure 7D). Next, we determined whether miR‐92b‐5p regulates intracellular lactate content by regulating MCT4 expression in C2C12 cells. Our results showed that MCT4 siRNA treatment increased the intracellular lactate content, which did not change after M92I5 treatment (Figure 7E). In contrast, MCT4 overexpression decreased the intracellular lactate content, which did not change after M92M5 treatment (Figure 7F). Taken together, we demonstrated that miR‐92b‐5p regulated lactate extrusion through inhibition of *MCT4* expression, by directly binding to the MCT4 3′‐UTR.

### UGP2 and MCT4 are required for glycogen induction and lactate extrusion from the skeletal muscles of M92KO mice

**Figure 8:**
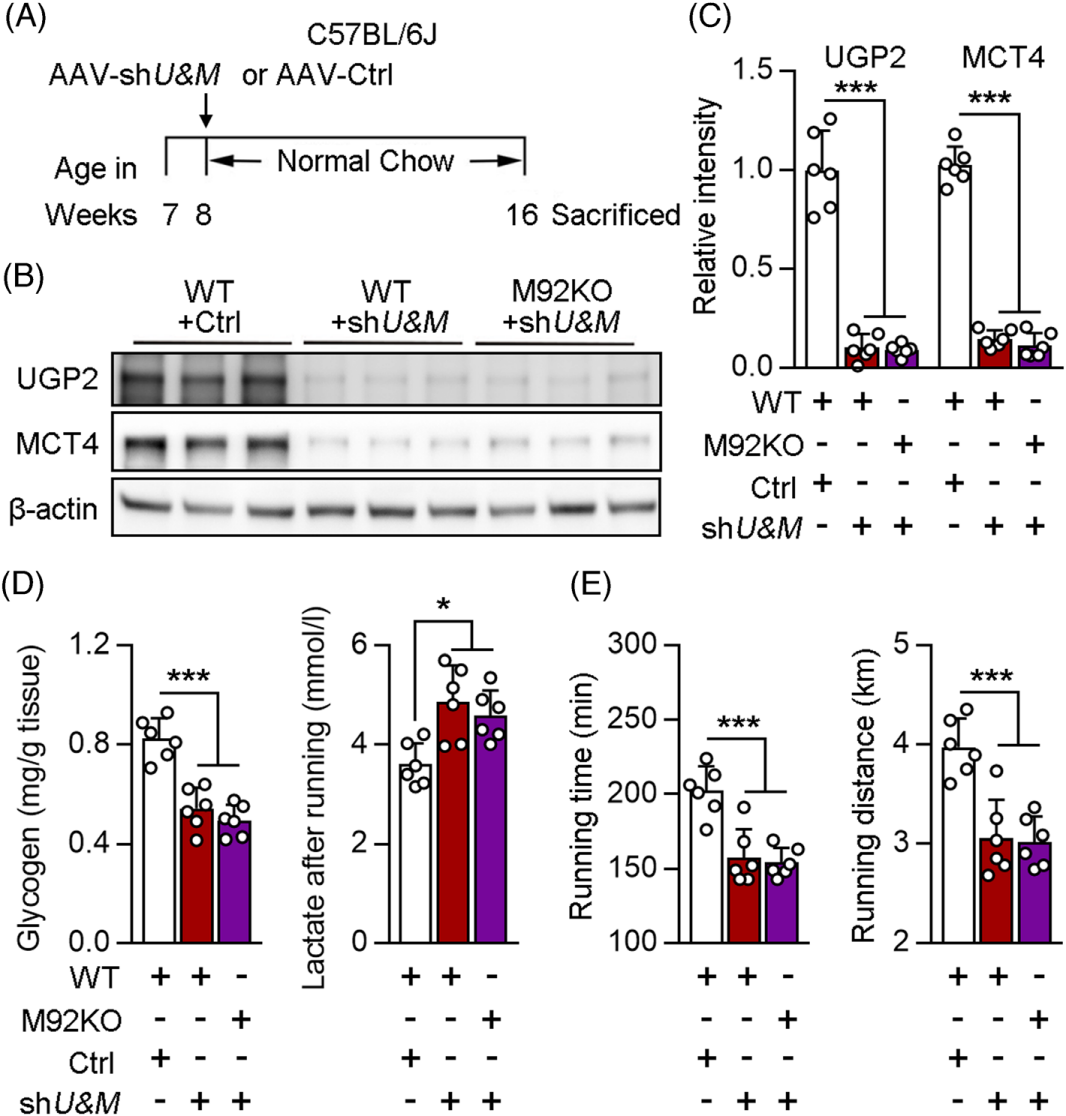
UGP2 and MCT4 mediates the effect of miR‐92b on exercise capacity, glycogen synthesis and lactate extrusion. (A to E) WT or M92KO mice transfected with AAV‐Ctrl or AAV‐shU&M (shUGP2 and shMCT4) as indicated (A), and western blot analysis of UGP2, MCT4 and β‐actin in Gas from mice (B), and the quantitative result were shown in the right panel (C). (D, E) The glycogen level of Gas from mice before running exhaustion (*n* = 6), the lactate level of Gas from mice after running exhaustion (*n* = 6), and the running time (left) and distance (middle) to exhaustion (*n* = 6). All results are expressed as means ± SD. *P < 0.05, **P < 0.01, ***P < 0.001, by a one‐way ANOVA with a Bonferroni correction test.

To further test whether UGP2 and MCT4 mediate the effect of muscle miR‐92b knockout on glycogen and lactate, AAV9‐hsa‐sh*Ugp2* was transfected into WT or M92KO mice (Figure S6A, B), with no significant differences observed in body weight (data not shown). When compared with the AAV‐Ctrl‐transfected WT mice, the AAV‐sh*Ugp2*‐transfected WT mice showed significantly decreased glycogen content before exercise exhaustion and reduced the exercise capacity (Figure S6C). Furthermore, the AAV‐sh*Ugp2*‐transfected M92KO mice had a lower lactate content in the Gas after exercise exhaustion and a higher exercise capacity than the AAV‐sh*Ugp2*‐transfected WT mice, but not glycogen content in the Gas before exercise exhaustion (Figure S6C). We knocked down MCT4 directly in the skeletal muscle of the mice using AAV9‐hsa‐sh*Mct4* delivery (Figure S6D, E), with no significant differences observed in body weight (data not shown). In the WT mice, sh*Mct4* transfection upregulated the lactate content of Gas after exercise and reduced the exercise capacity of the mice, but had no evident effect on the glycogen content of the Gas before exercise (Figure S6F). However, AAV‐sh*Mct4*‐transfected M92KO mice still showed a significant increase in the glycogen content of the Gas before exercise and exercise capacity than AAV‐sh*Mct4*‐transfected WT mice (Figure S6F). UGP2 and MCT4 protein levels in the Gas were significantly decreased in the AAV‐*shU&M* (AAV‐sh*Ugp2* and AAV‐sh*Mct4*)‐injected mice than in the AAV‐Ctrl‐injected mice (Figure 8A–C), with no significant differences observed in body weight (data not shown). There was no significant difference in glycogen content before exercise and lactate content after exercise in the Gas between WT and M92KO mice treated with AAV‐sh*U&M* (Figure 8D). More importantly, when UGP2 and MCT4 were absent, M92KO mice displayed exercise capacity that was similar to that of WT mice (Figure 8E), thereby suggesting that knockout of miR‐92b affects skeletal muscle functions mainly by regulating the expression of UGP2 and MCT4.

### Endurance training inhibits miR‐92b expression through negative regulation of hypoxia

A previous study has shown that miR‐92b is likely to be transcriptionally induced by hypoxia.^29^ Consistent with this, we found that both miR‐92b‐3p and miR‐92b‐5p were upregulated under hypoxia (Figure S7A). The transcription factor hypoxia‐inducible factor (*HIF*) has been suggested as a candidate for mediating training adaptation in skeletal muscles. After 4 weeks of unilateral knee‐extensor training, HIF‐1 mRNA levels transiently increased only in the untrained leg, when exposed to acute exercise.^30^ Another study showed that endurance training enhanced the expression of negative regulators of HIF, such as prolyl hydroxylase domain (PHD) enzymes (PHD 1, 2 and 3), which catalyse HIF prolyl hydroxylation, leading to the degradation of the oxygen sensors HIF‐1α and HIF‐2α.^31^ In agreement with this, we found that trained mice (4 weeks training) displayed higher protein level of PHD2 (the primary HIF‐1 PHD under normal conditions) in the Gas, and downregulated levels of HIF‐1α, than untrained mice (Figure S7B). In addition, PHD1 and PHD2 mRNA levels, but not PHD3 mRNA levels, increased after training (Figure S7C). Further analysis of miR‐92b expression revealed that trained mice displayed lower level of miR‐92b in the Gas than untrained mice (Figure S7D). The expression of miR‐92b‐3p increased sharply in the Gas from mice subjected to acute exercise, as compared with that in the no exercise mice, but returned to baseline after 120 min; long‐term training weakened these acute exercise‐induced effects (Figure S7D). Taken together, long‐term training inhibited miR‐92b expression by increasing the expression of the negative regulators of hypoxia.

## Discussion

In this study, we identified a novel role of miR‐92b in the skeletal muscle that affects physical activity. There is a decrease in miR‐92b levels in the skeletal muscle of humans and mice after exercise training for a certain period, but an increase after acute exercise. Mice lacking miR‐92b displayed a normal metabolic phenotype, but with induction of exercise capacity. Corroboratively, overexpression of miR‐92b in the skeletal muscle resulted in a similar metabolic phenotype and reduced exercise capacity.

Previous research has shown that a regulatory axis consisting of Akt signalling controls glycogen synthesis and glucose homeostasis during insulin signalling.^32^ As what mentioned before, miR‐92b is involved in the regulation of Akt signalling, both in vivo and in vitro. However, in our study, based on RNA‐seq data, we did not observe obvious changes in PTEN and Gabra3 expression in the miR‐92b‐ablated muscle. Moreover, Akt signalling in skeletal muscles can regulate skeletal muscle growth and atrophy.^23^ However, we did not observe altered expression of Akt signalling‐related genes (according to Wiki Pathway Analysis) or muscle hypertrophy in the M92KO mice, as compared with that in the controls.

Notably, a previous study found that inhibiting miR‐92 in skeletal muscle by antagomir failed to modulate skeletal anabolic response to mechanical loading.^33^ Consistent with these findings, we observed that M92KO had no significant effect on grip strength in mice (Figure 3A). Upon comparing the nucleotide sequences, we identified a discrepancy between the antagomir sequence (5′‐caggccgggacaagugcaau‐3′) and the miR‐92b‐5p sequence (5′‐agggacgggacguggugcaguguu‐3′). This suggests that the antagomir may be ineffective in repressing the expression of miR‐92b‐5p. Given the crucial role of miR‐92b‐5p in regulating lactate extrusion and exercise capacity (Figure 7F), it is plausible that this discrepancy could account for the different results observed in our study’. As miR17‐92 cluster (contains six miRs: 17, 18a, 19a, 20a, 19b and 92a) expressed in type I collagen‐producing cells is a key regulator of periosteal bone formation in mice,^34^ we next investigated the expression of other miRs from the miR‐17‐92 cluster following exercise/unloading. Interestingly, we found that miR‐19b mRNA levels were also higher in the Gas muscles from the hindlimb suspension (HLS) mice, but lower after 7 days of reloading (data not shown).

Through reanalysis of the database obtained from GSE209880,^35^ we found that both miR‐92b‐3p and miR‐92b‐5p expression in the skeletal muscle were increased after 24 h of a single exercise bout, as compared with baseline (Figure S7E). Consistently, we found that a single exercise bout upregulated the expression of both miR‐92b‐3p and miR‐92b‐5p in the skeletal muscle of mice (Figure S7D). In contrast, another study did not find a reduction in miR‐92b in skeletal muscle after 72 h of high aerobic exercise with sleep restriction.^36^ This discrepancy may be attributed to the influence of sleep quality on miR‐92b expression.^37^ Next, to further clarify whether long‐term exercise training decreases miR‐92b expression, we performed a reanalysis of the database obtained from right vastus lateralis biopsy specimens of patients with type 2 diabetes before and after 16 weeks of chronic exercise training (GSE58248). The results showed that miR‐92b expression in the vastus lateralis decreased after 12 weeks of aerobic exercise, but not resistance training exercise, compared with baseline (Figure S7F). These findings suggest that miR‐92b expression in skeletal muscle may be influenced by the type and duration of exercise training.

We demonstrated a miR‐92b‐mediated feedback signalling pathway between exercise and exercise‐induced exhaustion. When running, miR‐92b is induced, which inhibits UPG2‐associated glycogen synthesis, as well as MCT4‐mediated lactate extrusion, eventually leading to glycogen depletion and exhaustion. Ablation of miR‐92b facilitated glycogen synthesis, lactate extrusion and endurance exercise capacity in skeletal muscles without previous training. Thus, we propose that miR‐92b may play an important role in manipulating exercise fatigue.

## Conflict of interest

The authors have declared that no conflict of interest exists.

## Supporting information


**Table S1.** The sequences of primers for qPCR analysis.
**Table S2.** Antibodies Information.
**Figure S1.** Skeletal muscle weight in hindlimb suspension mice. (A, B) The weight of GAS and TA of WT mice in control, unload, and unload with reload groups (*n* = 6). All results are expressed as means ± SD. *p < 0.05, **p < 0.01, ***p < 0.001, by a one‐way ANOVA.
**Figure S2.** MiR‐92b knockout has no evident effect in blood glucose. (A) The qPCR was used to determine miR‐92b‐3p and miR‐92b‐5p in Gas muscle from WT and miR‐92b knockout (M92KO) mice, *n* = 6. (B) The ratio of heart weight (HW; mg) and tibia length (TL; mm) were shown in panel B (*n* = 6). (C) Daily food intake were shown in panel C (n = 6). (D) The fasting blood glucose and refed blood glucose in WT or M92KO mice (n = 6). All results are expressed as means ± SD. *p < 0.05, **p < 0.01, ***p < 0.001, by unpaired Student's t test.
**Figure S3.** MiR‐92b overexpression has no evident effect in blood glucose (A) The qPCR was used to determine miR‐92b‐3p and miR‐92b‐5p in Gas muscle from WT and miR‐92b overexpression (M92OE) mice, *n* = 6. (B) The fasting blood glucose, fasting blood insulin and refed blood glucose in WT or M92OE mice (n = 6). All results are expressed as means ± SD. *p < 0.05, **p < 0.01, ***p < 0.001, by unpaired Student's t test.
**Figure S4.** MiR‐92b knockout improves insulin resistance in diabetic mice. (A) WT or M92KO mice fed with HFD diet and treated with STZ to establish a diabetes model. (B) The qPCR was used to determine miR‐92b‐3p and miR‐92b‐5p in Gas muscle from WT and M92KO diabetic mice, *n* = 6. (C) The ratio of heart weight (HW; mg) and tibia length (TL; mm) were shown in left panel (*n* = 6). Daily food intake were shown in right panel (n = 6). (D) The fasting blood glucose, fasting blood insulin and refed blood glucose in diabetic mice (*n* = 6). (E) Western blot analysis of p‐Akt, Akt, p‐mTOR, mTOR, p‐S6K, S6K, and β‐actin were shown in the panel F, and the quantitative result were shown in the panel M (*n* = 6). (F) The mRNA level of *MuRF1* and *Atrogin‐1* was detected by qPCR in Gas muscle of diabetic mice (n = 6). All results are expressed as means ± SD. *p < 0.05, **p < 0.01, ***p < 0.001, by unpaired Student's t test.
**Figure S5.** MiR‐92b inhibits the protein and mRNA level of UGP2 and MCT4 *in vivo*.(A) Western blot analysis of UGP2 and β‐actin in Gas from WT, M92KO and M92OE mice (left panel), and the quantitative result were shown in the right panel (*n* = 3). (B) The qPCR was used to determine UGP2 in Gas muscle from WT, M92KO and M92OE mice (*n* = 6). (C) C2C12 cells treated with miR‐92b‐3p inhibitor (M92I) or Ctrl RNA, the protein level of UGP2 and β‐actin were determined by western blot and the intracellular glycogen was detected by commercial kit (*n* = 3). (D) C2C12 cells treated with M92I and/or *Ugp2* siRNA, the protein level of UGP2 and β‐actin were determined by western blot, and the intracellular glycogen was detected by commercial kit (*n* = 3). (E) Western blot analysis of MCT4 and β‐actin in Gas from WT, M92KO and M92OE mice (left panel), and the quantitative result were shown in the right panel (n = 3). (F) The qPCR was used to determine MCT4 in Gas muscle from WT, M92KO and M92OE mice (*n* = 6). All results are expressed as means ± SD. *p < 0.05, **p < 0.01, ***p < 0.001, by unpaired Student's t test (E to I). *p < 0.05, **p < 0.01, ***p < 0.001, by a one‐way ANOVA (J, K).
**Figure S6.** MiR‐92b regulates exercise capacity through UGP2 and MCT4 *in vivo*. (A to C) A, B:WT or M92KO mice transfected with AAV‐Ctrl or AAV‐shUGP2 as indicated and western blot analysis of UGP2, MCT4 and β‐actin in Gas from mice (left panel), and the quantitative result were shown in the right panel(*n* = 6). C: The glycogen level of Gas from mice before running exhaustion (n = 6), the lactate level of Gas from mice after running exhaustion (n = 6), and the running time (left) and distance (middle) to exhaustion (*n* = 6). (D to F) D, E: WT or M92KO mice transfected with AAV‐Ctrl or AAV‐shMCT4 as indicated and western blot analysis of UGP2, MCT4 and β‐actin in Gas from mice (left panel), and the quantitative result were shown in the right panel (*n* = 6). F: The glycogen level of Gas from mice before running exhaustion (n = 6), the lactate level of Gas from mice after running exhaustion (n = 6), and the running time (left) and distance (middle) to exhaustion (n = 6). All results are expressed as means ± SD. *p < 0.05, **p < 0.01, ***p < 0.001, by a one‐way ANOVA.
**Figure S7.** Exercise regulates the expression level of miR‐92b. (A) The protein level of HIF‐1α and β‐actin in C2C12 cells under normoxia and hypoxia, and the miR‐92b‐3p and miR‐92b‐5p was detected by qPCR (*n* = 3). (B, C) Western blot analysis of PHD2, HIF‐1α and β‐actin in Gas from untrained and trained mice (panel B), and the mRNA level of PHD1, PHD2, and PHD3 were determined by qPCR (panel C) (*n* = 6). (D) The expressions of miR‐92b‐3p and miR‐92b‐5p were detected at different time points after acute exercise (n = 3; noexercise mice was used as control). (E) Reanalyzed the database obtained from GEO database (GSE209880) on human specimens from skeletal muscle before and after a single exercise bout (*n* = 39). (F) Reanalysis of the database obtained from right vastus lateralis biopsy specimens of patients with type 2 diabetes before and after 16 weeks of chronic exercise training (GSE58248). All results are expressed as means ± SD. *p < 0.05, **p < 0.01, ***p < 0.001, by unpaired Student's t test.

**Data S1.** Supporting Information.

## Data Availability

Any additional information required to reanalyse the data reported in this paper is available from the lead contact upon request. The sequencing data reported in this paper are deposited in NCBI Gene Expression Omnibus (GEO) database with accession number GEO: GSE242294 and will be publicly available as of the date of publication.
