# Supplementary material for: MicroRNA‐92b in the skeletal muscle regulates exercise capacity via modulation of glucose metabolism
Source: J Cachexia Sarcopenia Muscle. 2023 Nov 20;14(6):2925–38. doi: 10.1002/jcsm.13377 (PMC10751421; doi:10.1002/jcsm.13377)

**Electronic Supplementary Materials**

**Supplementary Methods**

**2.1 Studies in animals**

All animal care and experimental protocols for *in vivo* studies conformed to the Guide for the Care and Use of Laboratory Animals published by the National Institutes of Health (NIH; NIH publication no.: 85–23, revised 1996). All experimental animal procedures were approved by the Ethics Committee of the Second Clinical Medical College of Jinan University **(The ethics approval number: 20210303-52)**, Shenzhen People’s Hospital (Shenzhen, China). All animals were randomized before treatment.

During treatment, the body weight of the mice was determined weekly and the average food intake was recorded daily. GTTs and ITTs were performed. After 8 weeks of treatment, the mice underwent skeletal muscle performance and endurance tests, and their blood glucose levels and body composition were measured (see below). Subsequently, all mice were allowed to fast overnight (16 h), anesthetized, and euthanized in a CO_2_ chamber, following which their blood and tissue samples were obtained for further analysis. Tibial bones were isolated from the mice, their hair rinsed off, and bone length measured using a ruler. Whole hearts from the mice were weighed and normalized to the tibia length. After treatment, all mice were anesthetized and euthanized in a CO_2_ chamber, after 16 h of fasting, following which their blood and muscle samples were harvested for further analysis.

**2.2 Exercise Protocol 1: Setting up the treadmill**

Prior to training and testing mice, we ensured that the treadmill was on a flat surface and set it to the desired angle of inclination, which was 0 degrees in our research. We set the electric shock frequency and intensity appropriately, which was 0.5 milliamp. We placed a piece of paper or an absorbent pad under the treadmill to collect feces and urine. Then, we placed a pad over one third of the treadmill housing furthest from the shock grid to create a darkened space and entice the mice to stay in that area.

**2.3 Exercise Protocol 2: Long-term training exercise**

We allowed a two-day acclimation period before initiating the training. The mice were gently placed onto the treadmill belt while it was turned off, and their corresponding shock grids were activated. The treadmill was gradually started, increasing the speed to a range of 1.5 to 3.0 meters per minute to encourage the mice to begin walking. Any mice that did not initiate walking or moved towards the shock grid were gently stimulated with a wire brush or their tails were tickled to encourage them to walk. After five minutes, the speed was increased to nine meters per minute, and after seven minutes, it was further increased to 10 meters per minute. The training session was concluded after 10 minutes by stopping the treadmill.

On the second day of training, the same procedure was followed, but the treadmill speed was set to 10 meters per minute from the beginning. The mice were loaded onto the treadmill, the shock grids were activated, and the treadmill was started. The training session lasted for 10 minutes.

To initiate the test phase, the treadmill and stopwatch were started simultaneously, and the speed was immediately set to 10 meters per minute. After 10 minutes, the speed was increased to 20 meters per minute, and the treadmill was stopped after 30 minutes. After four weeks of exercise, all mice were humanely euthanized in a CO2 chamber following a 16-hour fasting period. Blood and muscle samples were collected for further analysis.

**2.4 Exercise capacity measurement**

Mice at eight weeks of age were allowed an acclimation period of two days on the treadmill before the exercise test. In case of adaptive exercise, the treadmill was set without an incline, and the exercise began with a 10 min acclimation period at 10 m·min^−1^, followed by 50 min at 20 m·min^−1^. In case of the exercise test, the treadmill was set without an incline, and the exercise started off at a speed of 10 m·min^−1^ for 10 min, followed by an increase to 20 m·min^−1^ until the mice were exhausted (which was characterized by the mice spending more than 5 s on the electric shocker without resuming running). The running time and distance till exhaustion were then measured. As for diabetic mice, the treadmill was set without an incline, and the exercise began with a 10 min acclimation period at 5 m·min^−1^, followed by an increase to 10 m·min^−1^ until the mice were exhausted.

**2.5 Suspension test**

The mice were randomly divided into control (non-suspension), unload (suspension for 7 d), and reload (7 d reload after 7 d suspension) groups, to generate the HLS model described previously[1]. Briefly, 10-week-old male mice were subjected to HLS for 7 d. The reloaded mice were suspended for 7 d and then reloaded for another 7 d. The weight of the skeletal muscle was measured after the mice were sacrificed.

**2.6 Analysis of miR-92b levels**

MiR-92b-3p and miR-92b-5p primers (Tsingke, Beijing, China) were used to determine the miR-92b-3p and miR-92b-5p levels in mice tissues (TA, Gas, soleus muscle, fat, and heart) samples. Tissue miR-92b-3p and miR-92b-5p levels were normalized to those of miR-191-5p, based on the stable expression across different cells and tissues of miR-191-5p[2]. The primers used for quantitative RT-PCR were as follows: 1) miR-92b-3p primers[3]: forward 5′-gtccgctattgcactcgtcccggcctcc-3′ and reverse 5′-gtgcgtgtcgtggagtc-3′; 2) miR-92b-5p primers[4]: forward 5′-agggacgggacgcggtgcagtg-3′ and reverse 5′-gcgagcacagaattaatacgac-3′; 3) miR-423-5p primers[5]: forward 5’- cagtgcgtgtcgtggagt-3’ and reverse 5’-gccctgaggggcagagagc-3’; 4) miR-191-5p primers[6]: forward 5’- acactccagctgggcaacggaatcccaaaag-3’ and reverse 5’- tggtgtcgtggagtcg-3’.

**2.7 Body composition measurements**

Total fat and lean mass were analyzed using a fully automatic EchoMRI™ system (Echo Medical Systems, Texas, USA), according to the manufacturer’s instructions[7].

**2.8 Histological analysis**

For H&E staining, the skeletal muscles of male mice were harvested, fixed in 4% paraformaldehyde (Sigma-Aldrich, St. Louis, MO, USA), and embedded in paraffin wax. Sections (5 µm) were cut and stained with a H&E kit (Solarbio Science & Technology, Beijing, China), using a standard protocol. For ATPase staining, the skeletal muscles of male mice were harvested and frozen in OCT, and 8-μm thick serial sections were cut using a freezing microtome (Leica Biosystems, Wetzlar, Germany), for staining. ATPase staining was performed using the ATPase Stain Kit (calcium-cobalt method) (Solarbio Science & Technology), as a standard procedure. Briefly, sections were cut and pre-incubated for 15 min, at pH 4.5, and incubated for 45 min, at pH 9.4, for the standard ATPase reaction.

**2.9 Determination of serum glucose and insulin levels**

Serum samples from miR-92b KO or AAV-hsa-M92OE-contrast WT mice were used to measure blood glucose and insulin levels, using a OneTouch^®^ glucometer and test strips (LifeScan, Milpitas, CA, USA) or an insulin ELISA kit (ab277390; Abcam, Cambridge, MA, USA), respectively. For refed glucose samples, the miR-92b KO or AAV-hsa-M92OE-contrast WT mice were allowed to fast overnight, following which their food was replaced for 2 h before determination of refed serum glucose levels. Serum insulin levels were determined after treatment, using a mouse insulin ELISA kit (ab277390) obtained from Abcam.

**2.10 GTT and ITT**

GTT and ITT were performed in mice, at 14 and 15 weeks of age, respectively. Briefly, mice were allowed to fast for 6 h and then intraperitoneally injected with glucose (2 g glucose/kg body weight; Y0001745, Sigma-Aldrich) for the GTT assay, or insulin (1 U insulin/kg body weight; Actrapid, Novo Nordisk, Denmark) for the ITT assay. Glucose levels were determined at 0, 15, 30, 60, 90, and 120 min after injection. During the treatment, fasting blood glucose was determined in the blood sample withdrawn from the mouse tail vein, using the OneTouch^®^ glucometer and test strips.

**2.11 Analysis of glycogen and lactate contents**

Glycogen and lactate contents in the mouse skeletal muscle were assessed using a Glycogen Assay Kit (ab65620, Abcam) and Lactate Assay Kit (ab169558, Abcam), respectively, according to the manufacturer’s instructions.

**2.12 Quantitative real-time PCR analysis**

All reagents were obtained from Sigma-Aldrich, except where indicated. Total RNA was extracted from cells or tissues using RNAiso Plus. The oligonucleotide primer sequences, synthesized by Generay (Shanghai, China), are listed in **Table 1**. The relative mRNA expression levels were normalized to the loading control (actin) using Quantity One^®^ Software (Bio-Rad Laboratories, California, USA).

**2.13 Protein extraction and western blot analysis**

Proteins from C2C12 cells or tissues obtained from mouse muscle (Gas) were extracted using radioimmunoprecipitation assay buffer (Solarbio Science & Technology) containing protease and phosphatase inhibitors (Thermo Fisher Scientific, Massachusetts, USA). The protein concentration was quantified using the Rapid Gold BCA Protein Assay Kit (Thermo Fisher), according to the manufacturer’s instructions. For western blot analysis, 50 µg of lysate was loaded onto sodium dodecyl sulphate-polyacrylamide gel electrophoresis gels, blotted onto polyvinylidene difluoride membranes (Millipore), and incubated with antibodies against: β-actin (ab213262), mTOR (ab25880), phosphorylated (p)-mTOR (Ser2448; ab109268), Akt (ab8805), p-Akt (Ser473; ab81283), GLUT1/UGP2 (ab252403), PHD2 (ab133630) and HIF-1α (ab221610) (all from Abcam), SLC16A3/MCT4 (NBP3-13033) (from Novus, Ontario, Canada), and p-p70 S6 kinase (Thr389) (9205S), p70 S6 kinase (9202S) (all from Cell Signaling Technology, Massachusetts, USA). Densitometry analysis was performed using Quantity One^®^ Software and quantified relative to the loading control, β-actin.

**2.14 Predicted targets of miR-92b**

Prediction of the target genes of differentially expressed miRNAs was accomplished by TargetScan (v8.0). **The Context++ score percentile is the comprehensive score of the predicted target, and the higher the score, the greater the probability that the predicted site is the real target. Hence, targeted genes with a Context++ score percentile less than 50, obtained with the TargetScan algorithm, were filtered and removed, because these predicted target genes are less likely to be real targets of miRNA.**

**2.15 Cell culture**

C2C12 myoblasts (SCSP-505) were purchased from the National Collection of Authenticated Cell Culture (Shanghai, China). Cells were regularly checked for mycoplasma in a standardized manner, using a qPCR test performed under ISO17025 accreditation, to ensure that the work was conducted in mycoplasma-negative cells. C2C12 myoblasts were cultured in growth medium (Dulbecco’s modified Eagle’s medium, high glucose; Gibco, Grand Island, NY, USA) containing 10% (vol./vol.) fetal bovine serum (Gibco), 10 U/mL penicillin, and 10 μg/mL streptomycin (Welgene, Taipei, China), at 37°C, in an incubator containing 5% CO_2_.

**2.16 C2C12 transient transfection**

The miR-92b-3p mimic was a duplex RNA, with the sense sequence 5′-uauugcacucgucccggccucc-3′ and antisense sequence 5′- aggccgggacgagugcaauauu-3′. The miR-92b-5p mimic was a duplex RNA with the sense sequence 5′-agggacgggacguggugcaguguu-3′ and antisense sequence 5′-aauauugcacuacgucccggcccu-3′. Non-targeting negative control sequences (sense 5′-uucuccgaacgugucacgutt-3′ and antisense 5′-acgugacacguucggagaatt-3′) were used as controls. The inhibitor of miR-92b-3p (5′-ggaggccgggacgagugcaaua-3′) is a single RNA sequence complementary to miR-92b-3p. The inhibitor of miR-92b-5p (5′-aacacugcaccacgucccgucccu-3′) is a single RNA sequence complementary to miR-92b-5p. A non-targeting negative control sequence (5′-caguacuuuuguguaguacaa-3′) was used as the control. siRNAs against Ugp2 (sc-154894), Mct4 (sc-40120), and scrambled siRNA were purchased from Santa Cruz Biotechnology (California, USA). Transient transfections were performed using Lipofectamine™ 3000 (Invitrogen), according to the manufacturer’s protocol.

**2.17 Dual-Luciferase**^®^ **Reporter Assay**

C2C12 cells were transfected with 3′-UTR luciferase reporter constructs (Ugp2 3′-UTR or Ugp2 3′-UTR-mutant), miRNA (control RNA [sc-36869; Santa Cruz Biotechnology] or miR-92b-3p), 3′-UTR luciferase reporter constructs (Mct4 3′-UTR or Mct4 3′-UTR-mutant), or miRNA (control RNA or miR-92b-5p), and Renilla luciferase, using Lipofectamine™ 3000. After 48 h of transfection, the luciferase activity was measured using a Dual-Luciferase^®^ Reporter Assay Kit (E1910; Promega, Madison, WI, USA) and a microplate reader (H1; Biotek, Winooski, VT, USA). Renilla luciferase was used to normalize the values.

**2.18 RNA Seq was analyzed by R software**

1. **Data processing**

**rt=as.matrix(rt)**

**rownames(rt)=rt[,1]**

**exp=rt[,2:ncol(rt)]**

**dimnames=list(rownames(exp),colnames(exp))**

**rt=matrix(as.numeric(as.matrix(exp)),nrow=nrow(exp),dimnames=dimnames)**

**rt=avereps(rt)**

1. **Data calibration**

**rt=normalizeBetweenArrays(as.matrix(rt))**

**rt=log2(rt+1)**

1. **Differential analysis**

**modType=c(rep("control", controlNum),rep("test",testNum))**

**design <- model.matrix(~0+factor(modType))**

**colnames(design) <- c("control","test")**

**fit <- lmFit(rt,design)**

**cont.matrix<-makeContrasts(test-control,levels=design)**

**fit2 <- contrasts.fit(fit, cont.matrix)**

**fit2 <- eBayes(fit2)**

**allDiff=topTable(fit2,adjust='fdr',number=200000)**

**Supplementary Table**

**Table S1** The sequences of primers for qPCR analysis

| Gene | Forward | Backward |
| --- | --- | --- |
| *mus-Actc1*  (ID: 11464) | CTGGATTCTGGCGATGGTGTA | CGGACAATTTCACGTTCAGCA |
| *mus-Pgc1α*  (ID: 19017) | TTCATCTGAGTATGGAGTCGCT | GGGGGTGAAACCACTTTTGTAA |
| *mus-Tnnc1*  (ID: 21924 ) | GCGGTAGAACAGTTGACAGAG | CCAGCTCCTTGGTGCTGAT |
| *mus-Pgc1β*  (ID: 170826) | TCCTGTAAAAGCCCGGAGTAT | GCTCTGGTAGGGGCAGTGA |
| *mus-MyHC IIa*  (ID: 17882) | AAGTGACTGTGAAAACAGAAGCA | GCAGCCATTTGTAAGGGTTGAC |
| *mus-MyHC IIb*  (ID: 17884) | TTGAAAAGACGAAGCAGCGAC | AGAGAGCGGGACTCCTTCTG |
| *mus-Tnni2*  (ID: 21953) | AGAGTGTGATGCTCCAGATAGC | AGCAACGTCGATCTTCGCA |
| *mus-Tnnc2*  (ID: 21925) | GAGGCCAGGTCCTACCTCAG | GGTGCCCAACTCTTTAACGCT |
| *mus-Ugp2*  (ID: [216558](http://www.ncbi.nih.gov/entrez/query.fcgi?db=gene&cmd=Retrieve&dopt=summary&list_uids=216558)) | AGCAAAGCTATGTCTCAAGATGG | GAGGCTGCTGTGGTAAGTATTT |
| *mus-Mct4*  (ID: 80879 ) | TCACGGGTTTCTCCTACGC | GCCAAAGCGGTTCACACAC |
| *mus-Phd1*  (ID: 112406) | TGGCCCTGGACTATATTGTGC | GGCTCGTGACCTTCTACCC |
| *mus-Phd2*  (ID: 112405) | GCCGCAGCTCCTTCTACTG | TTCATGCACGGCACGATGTA |
| *mus-Phd3*  (ID: 112407) | AGCGGTCCAAGGTAGGATG | ACACTCCGAATGAAGGCTTCC |
| *mus-β-actin*  (ID: 11461) | GGCTGTATTCCCCTCCATCG | CCAGTTGGTAACAATGCCATGT |

**Table S2 Antibodies Information**

| Antibodies | Source | Catalog No. | Antibody validation and specificity |
| --- | --- | --- | --- |
| Akt (1:1000) | Cell Signaling Technology | Cat# 2938S | **Validated by KO (Official verification, https://www.cellsignal.cn/products/primary-antibodies/akt1-c73h10-rabbit-mab/2938?site-search-type=Products&N=4294956287&Ntt=akt1&fromPage=plp)** |
| pAkt (1:1000) | Abcam | Cat#ab81283; RRID:AB_2224551 | **Validated by platelet derived growth factor, insulin or alkaline phosphatase treatment (****Official verification,** **https://www.abcam.cn/products/primary-antibodies/akt1-phospho-s473-antibody-ep2109y-ab81283.html#lb)** |
| UGP2 (1:1000) | Santa Cruz Biotechnology | Cat# sc-377089 | **Validated by OE (Official verification, https://www.scbt.com/zh/p/ugp2-antibody-b-3?requestFrom=search)** |
| SLC16A3/MCT4 (1:1000) | Novus Biologicals | Cat# NBP3-13033 | **Validated by KD or OE in the present manuscript**  **(Fig. 7E, F and 8B)** |
| p70 S6 kinase (1:1000) | Cell Signaling Technology | Cat# 9202S | **Validated by KD** **in previous study[8]** |
| Phospho  p70 S6 Kinase (Thr389) (1:1000) | Cell Signaling Technology | Cat# 97596S | **Validated by insulin like growth factor 1 or alkaline phosphatase treatment (Official verification,** **https://www.cellsignal.cn/products/primary-antibodies/phospho-p70-s6-kinase-thr389-d5u1o-rabbit-mab/97596?site-search-type=Products&N=4294956287&Ntt=s6+kinase&fromPage=plp)** |
| mTOR (1:1000) | Abcam | Cat#ab25880; RRID:AB_470770 | **Validated by mTOR OE in previous study[9]** |
| Phospho  mTOR (1:1000) | Abcam | Cat#ab109268;RRID:AB_10888105 | **Validated by alkaline phosphatase treatment (Official verification, https://www.abcam.cn/products/primary-antibodies/mtor-phospho-s2448-antibody-epr4262-ab109268.html)** |
| PHD2 (1:1000) | Abcam | Cat# ab133630 | **Validated by KO (Official verification, https://www.abcam.cn/products/primary-antibodies/phd2--prolyl-hydroxylase-antibody-epr3660b2-ab133630.html)** |
| β-actin (1:1000) | Abcam | Cat#ab213262;RRID:AB_10855480 | **Validated by KO (Official verification, https://www.abcam.cn/products/primary-antibodies/beta-actin-antibody-epr21241-ab213262.html)** |

**Abbreviations: KD, knockdown; KO, knockout; OE, overexpression**

**Supplementary Figure and Figure legend**


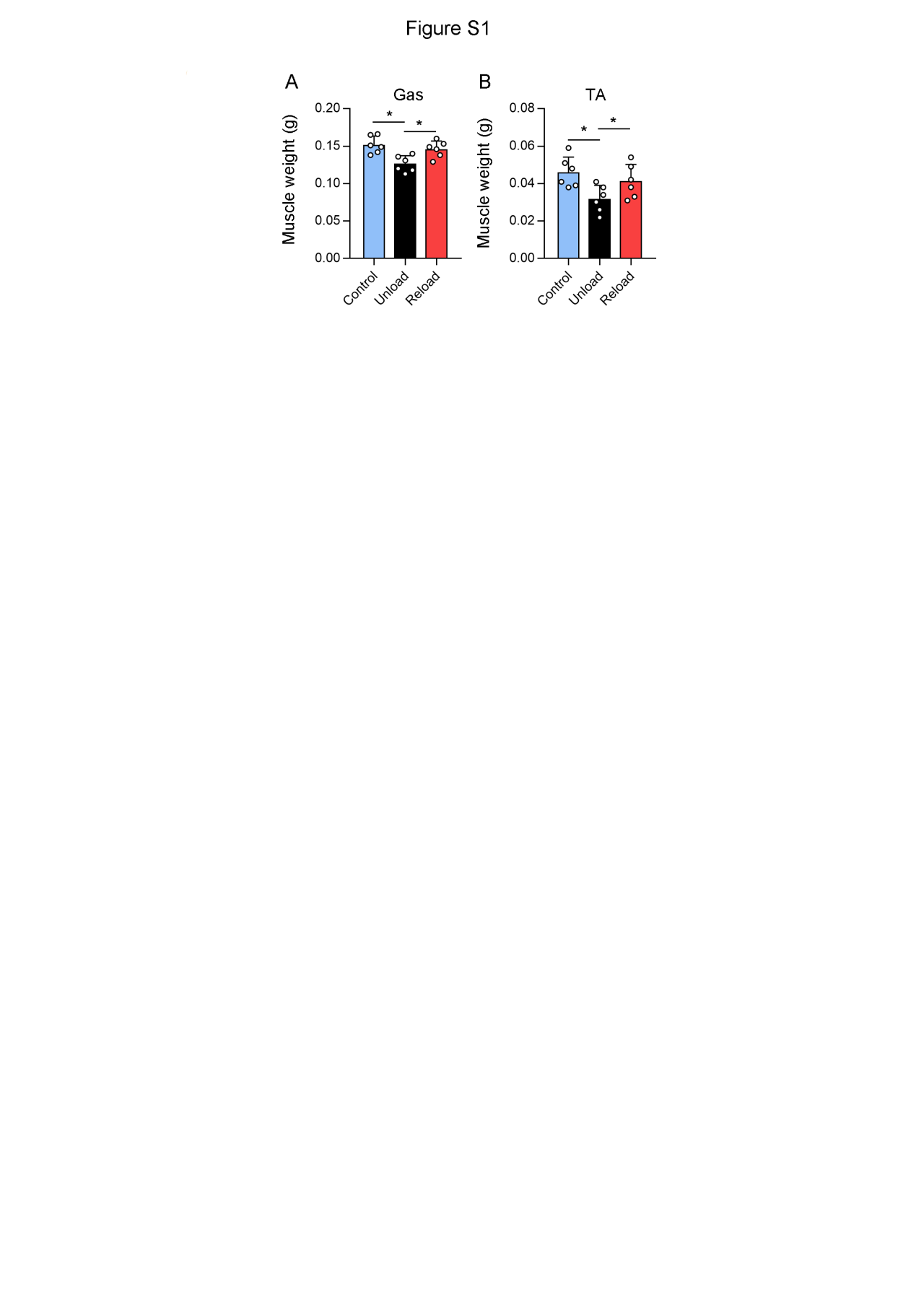


**Figure S1. Skeletal muscle weight in hindlimb suspension mice.** (A, B) The weight of GAS and TA of WT mice in control, unload, and unload with reload groups (n = 6). All results are expressed as means ± SD. **p*< 0.05, ***p* < 0.01, ****p* < 0.001, by a one-way ANOVA.


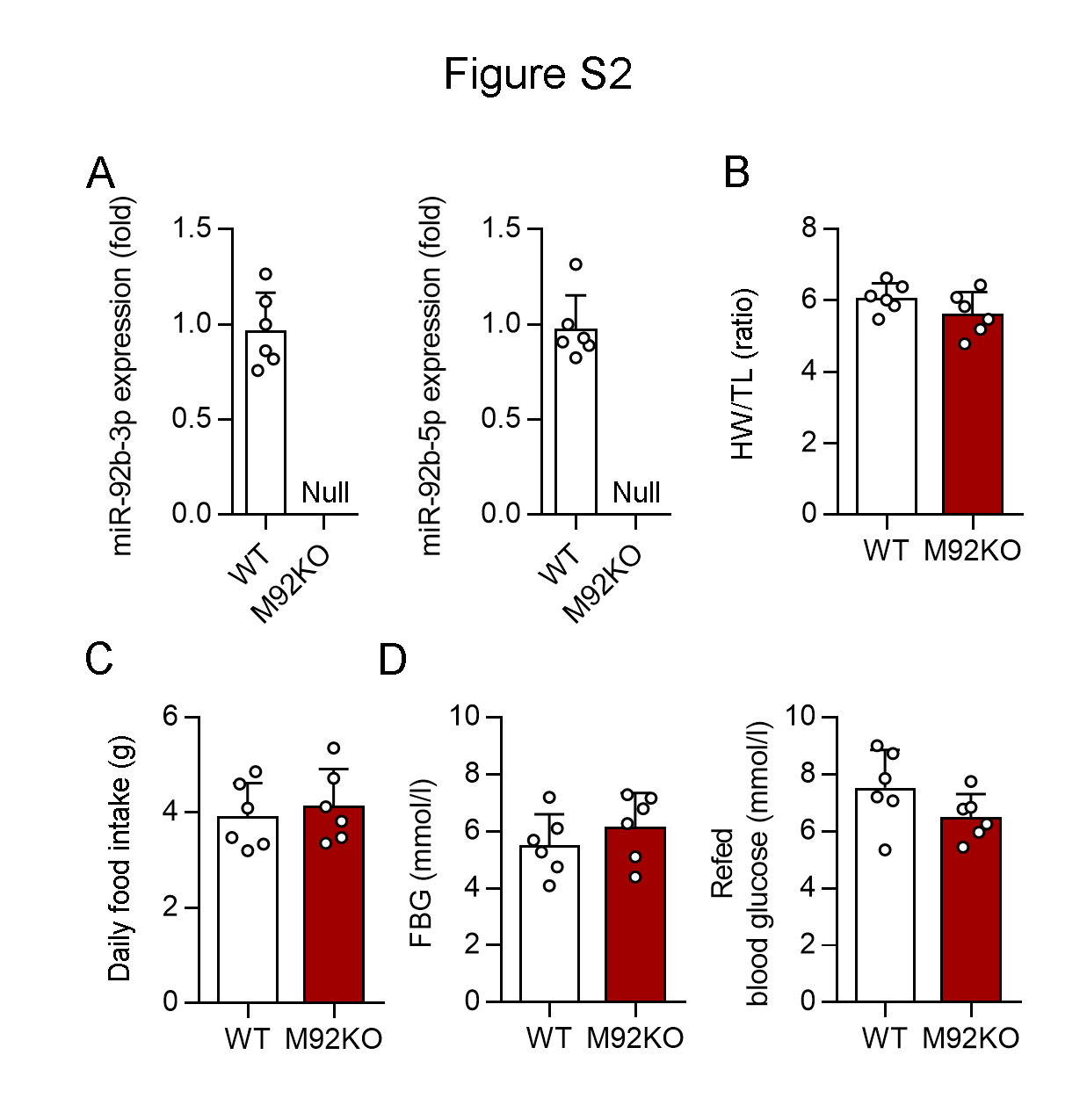


**Figure S2. MiR-92b knockout has no evident effect in blood glucose. (A)** The qPCR was used to determine miR-92b-3p and miR-92b-5p in Gas muscle from WT and miR-92b knockout (M92KO) mice, n = 6. (B) The ratio of heart weight (HW; mg) and tibia length (TL; mm) were shown in panel B (n = 6). (C) Daily food intake were shown in panel C (n = 6). (D) The fasting blood glucose and refed blood glucose in WT or M92KO mice (n = 6). All results are expressed as means ± SD. **p*< 0.05, ***p* < 0.01, ****p* < 0.001, by unpaired Student’s t test.


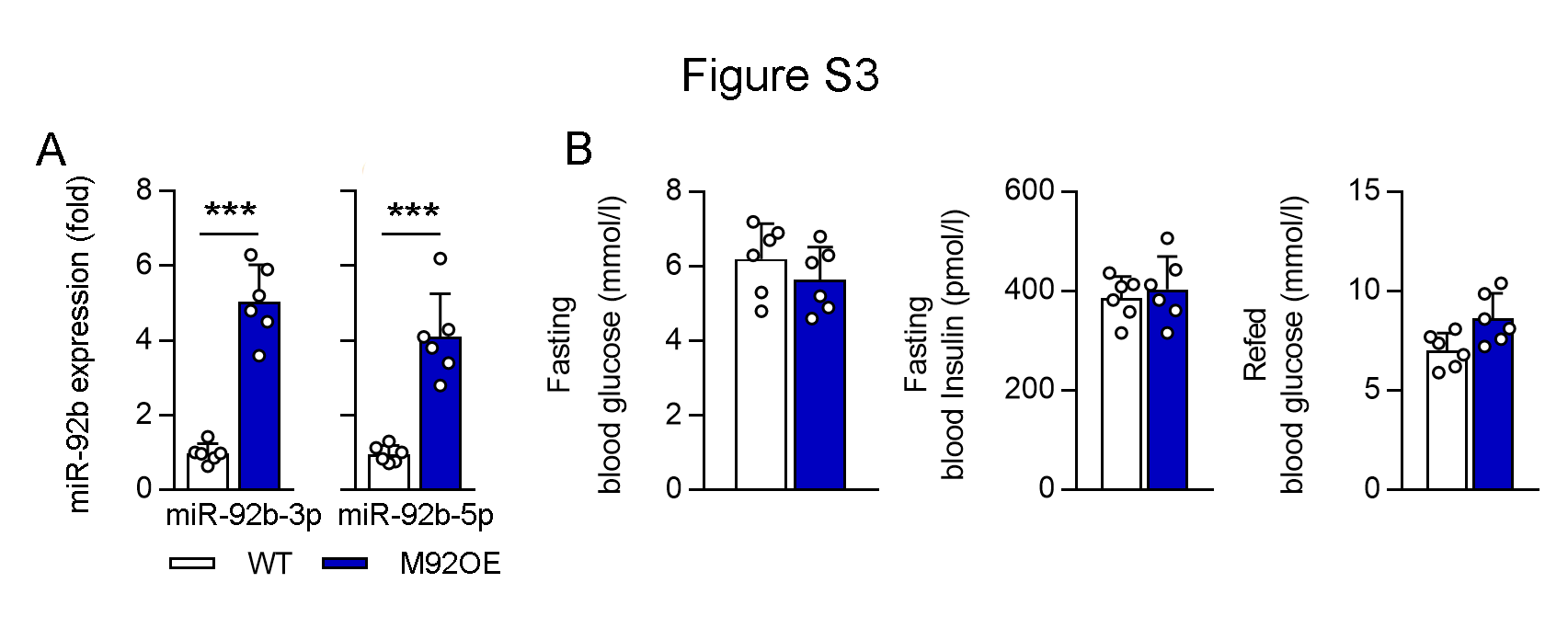


**Figure S3. MiR-92b overexpression has no evident effect in blood glucose (A)** The qPCR was used to determine miR-92b-3p and miR-92b-5p in Gas muscle from WT and miR-92b overexpression (M92OE) mice, n = 6. **(B)** The fasting blood glucose, fasting blood insulin and refed blood glucose in WT or M92OE mice (n = 6). All results are expressed as means ± SD. **p*< 0.05, ***p* < 0.01, ****p* < 0.001, by unpaired Student’s t test.


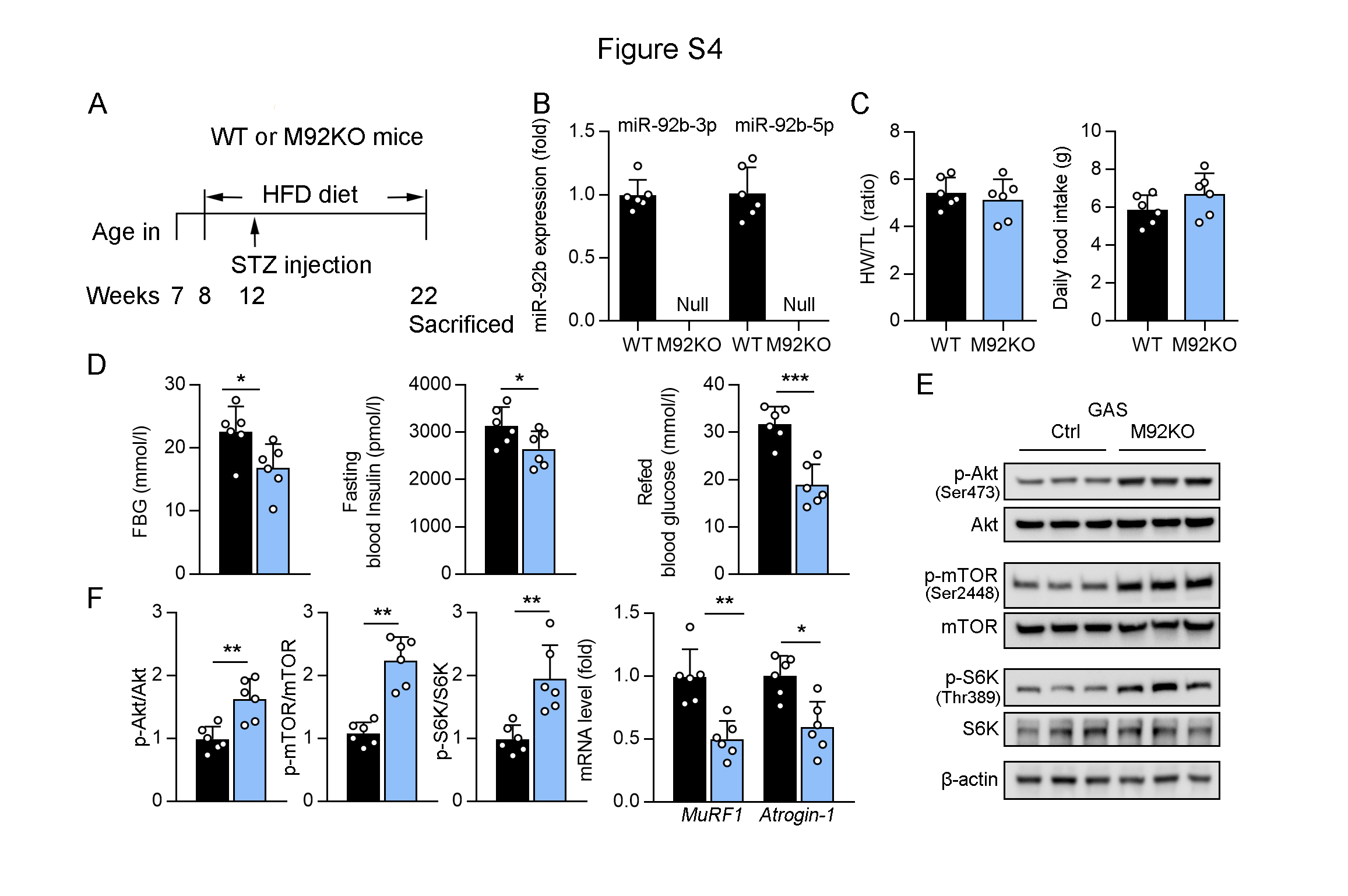


**Figure S4. MiR-92b knockout improves insulin resistance in diabetic mice. (A)** WT or M92KO mice fed with HFD diet and treated with STZ to establish a diabetes model. **(B)** The qPCR was used to determine miR-92b-3p and miR-92b-5p in Gas muscle from WT and M92KO diabetic mice, n = 6. **(C)** The ratio of heart weight (HW; mg) and tibia length (TL; mm) were shown in left panel (n = 6). Daily food intake were shown in right panel (n = 6). **(D)** The fasting blood glucose, fasting blood insulin and refed blood glucose in diabetic mice (n = 6). **(E)** Western blot analysis of p-Akt, Akt, p-mTOR, mTOR, p-S6K, S6K, and β-actin were shown in the panel F, and the quantitative result were shown in the panel M (n = 6). **(F)** The mRNA level of *MuRF1* and *Atrogin-1* was detected by qPCR in Gas muscle of diabetic mice (n = 6). All results are expressed as means ± SD. **p*< 0.05, ***p* < 0.01, ****p* < 0.001, by unpaired Student’s t test.


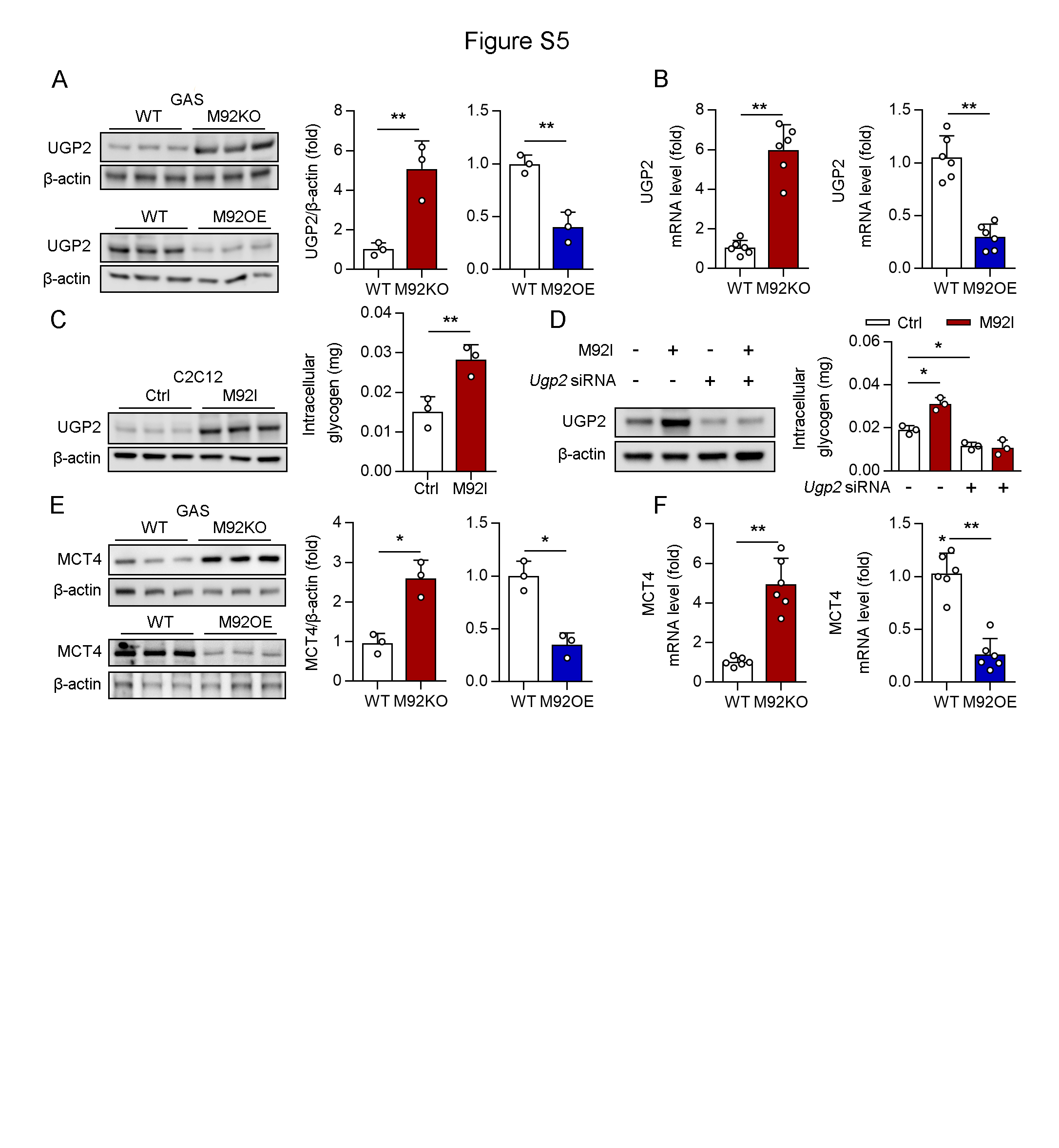


**Figure S5. MiR-92b inhibits the protein and mRNA level of UGP2 and MCT4 *in vivo*.(A)** Western blot analysis of UGP2 and β-actin in Gas from WT, M92KO and M92OE mice (left panel), and the quantitative result were shown in the right panel (n = 3). **(B)** The qPCR was used to determine UGP2 in Gas muscle from WT, M92KO and M92OE mice (n = 6). **(C)** C2C12 cells treated with miR-92b-3p inhibitor (M92I) or Ctrl RNA, the protein level of UGP2 and β-actin were determined by western blot and the intracellular glycogen was detected by commercial kit (n = 3). **(D)** C2C12 cells treated with M92I and/or *Ugp2* siRNA, the protein level of UGP2 and β-actin were determined by western blot, and the intracellular glycogen was detected by commercial kit (n = 3). **(E)** Western blot analysis of MCT4 and β-actin in Gas from WT, M92KO and M92OE mice (left panel), and the quantitative result were shown in the right panel (n = 3). **(F)** The qPCR was used to determine MCT4 in Gas muscle from WT, M92KO and M92OE mice (n = 6). All results are expressed as means ± SD. **p*< 0.05, ***p* < 0.01, ****p* < 0.001, by unpaired Student’s t test (E to I). **p*< 0.05, ***p* < 0.01, ****p* < 0.001, by a one-way ANOVA (J, K).


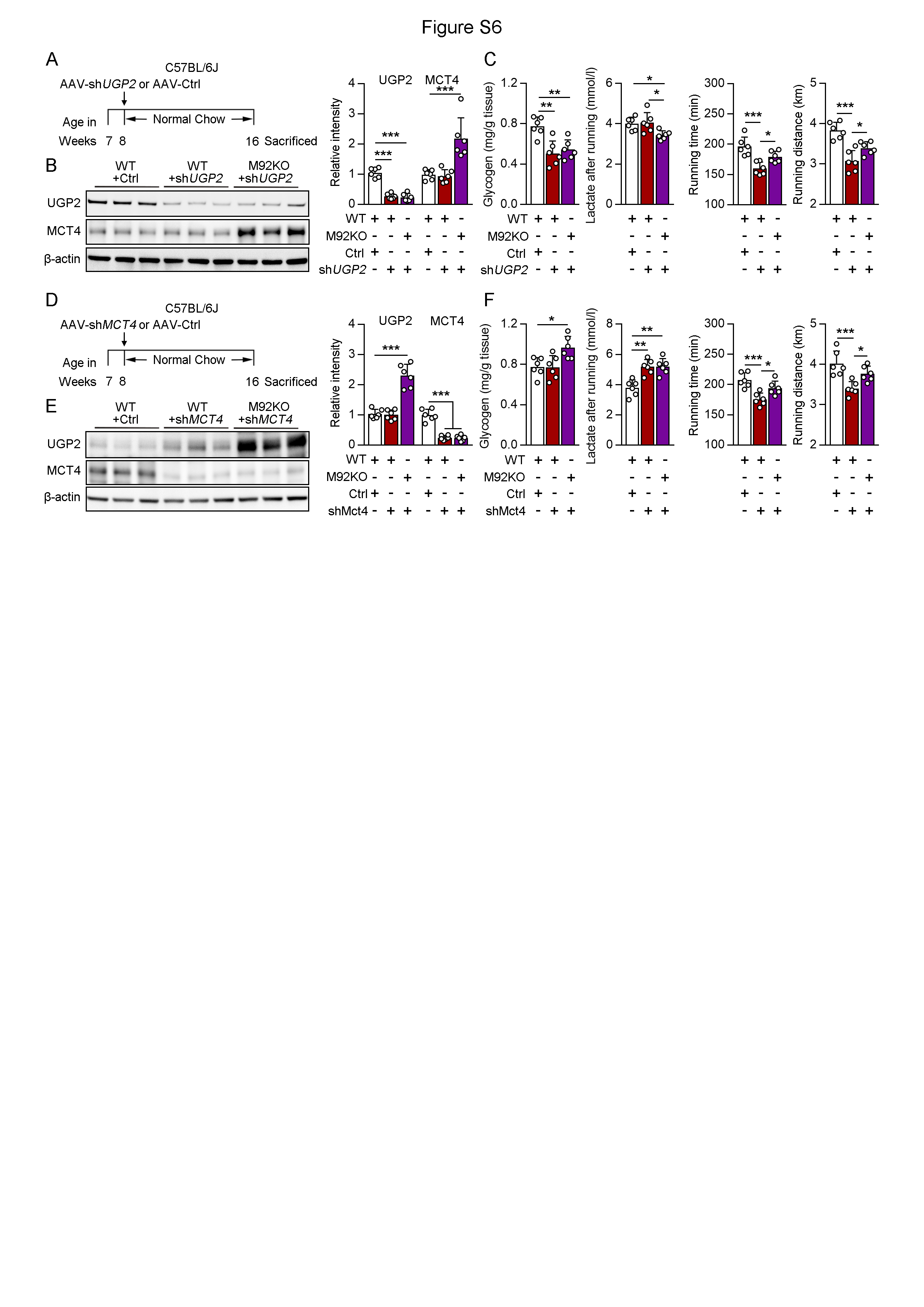


**Figure S6. MiR-92b regulates exercise capacity through UGP2 and MCT4 *in vivo*. (A to C) A, B:**WT or M92KO mice transfected with AAV-Ctrl or AAV-shUGP2 as indicated and western blot analysis of UGP2, MCT4 and β-actin in Gas from mice (left panel), and the quantitative result were shown in the right panel(n = 6). C: The glycogen level of Gas from mice before running exhaustion (n = 6), the lactate level of Gas from mice after running exhaustion (n = 6), and **t**he running time (left) and distance (middle) to exhaustion (n = 6). **(D to F)** D, E: WT or M92KO mice transfected with AAV-Ctrl or AAV-shMCT4 as indicated and western blot analysis of UGP2, MCT4 and β-actin in Gas from mice (left panel), and the quantitative result were shown in the right panel (n = 6). F: The glycogen level of Gas from mice before running exhaustion (n = 6), the lactate level of Gas from mice after running exhaustion (n = 6), and **t**he running time (left) and distance (middle) to exhaustion (n = 6). All results are expressed as means ± SD. **p*< 0.05, ***p* < 0.01, ****p* < 0.001, by a one-way ANOVA.


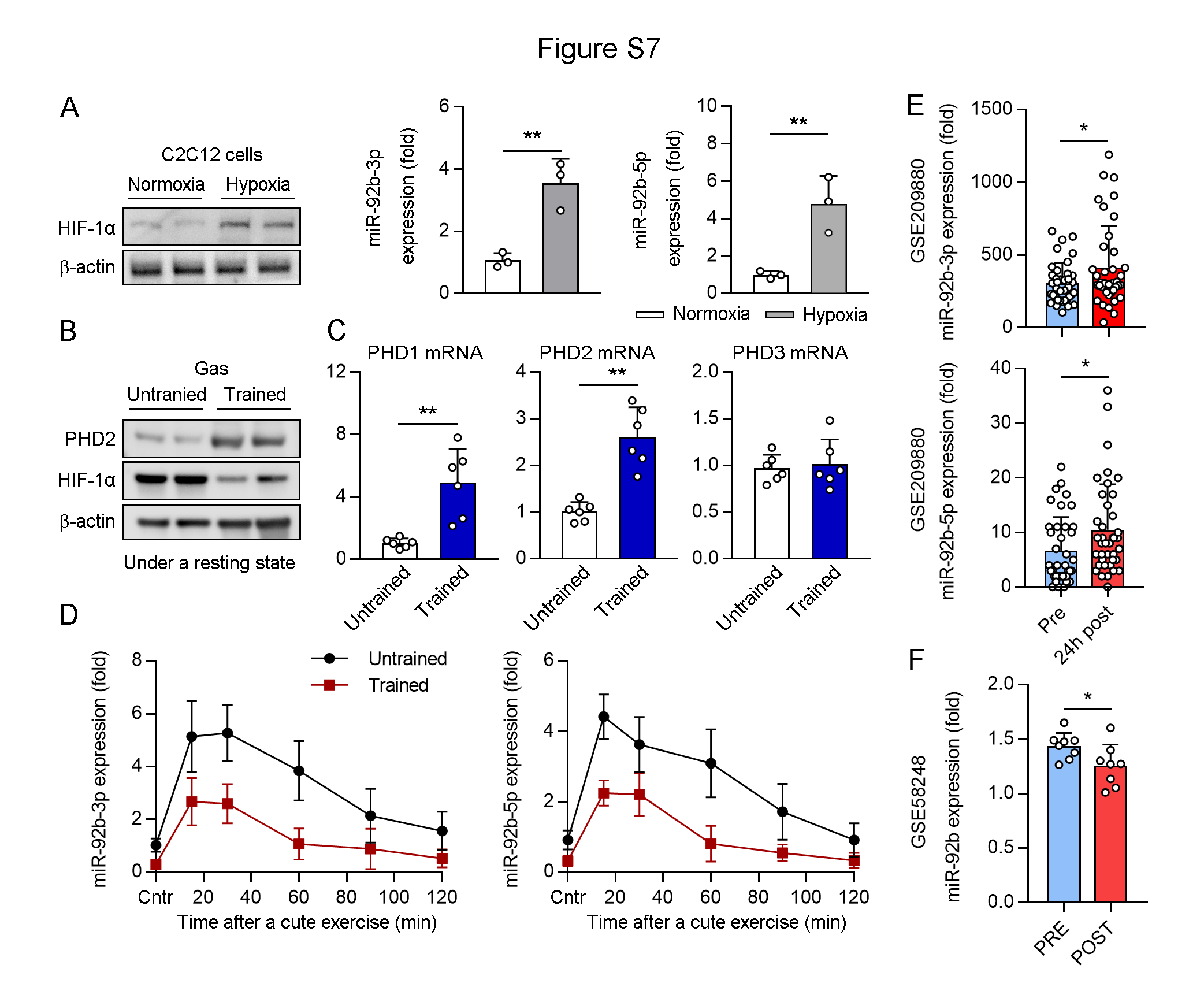


**Figure S7. Exercise regulates the expression level of miR-92b. (A)** The protein level of HIF-1α and β-actin in C2C12 cells under normoxia and hypoxia, and the miR-92b-3p and miR-92b-5p was detected by qPCR (n = 3). **(B, C)** Western blot analysis of PHD2, HIF-1α and β-actin in Gas from untrained and trained mice (panel B), and the mRNA level of PHD1, PHD2, and PHD3 were determined by qPCR (panel C) (n = 6). **(D)** The expressions of miR-92b-3p and miR-92b-5p were detected at different time points after acute exercise (n = 3; noexercise mice was used as control). **(E)** Reanalyzed the database obtained from GEO database (GSE209880) on human specimens from skeletal muscle before and after a single exercise bout (n = 39). **(F)** Reanalysis of the database obtained from right vastus lateralis biopsy specimens of patients with type 2 diabetes before and after 16 weeks of chronic exercise training (GSE58248). All results are expressed as means ± SD. **p*< 0.05, ***p* < 0.01, ****p* < 0.001, by unpaired Student’s t test.

**Reference**

1. Ferreira J, Crissey J, Brown M. An alternant method to the traditional NASA hindlimb unloading model in mice. Journal of visualized experiments : JoVE. 2011;

2. Russell A, Ghobrial L, Ngo S, Yerbury J, Zacharewicz E, Chung R, et al. Dysregulation of microRNA biogenesis machinery and microRNA/RNA ratio in skeletal muscle of amyotrophic lateral sclerosis mice. Muscle & nerve. 2018;57:838-47.

3. Huang Y, Tang J, Li X, Long X, Huang Y, Zhang X. miR-92b-3p Exerts Neuroprotective Effects on Ischemia/Reperfusion-Induced Cerebral Injury via Targeting NOX4 in a Rat Model. Oxidative medicine and cellular longevity. 2022;2022:3494262.

4. Lin X, Zhu J, Wang L, Yan F, Sha W, Yang H. MiR-92b-5p inhibitor suppresses IL-18 mediated inflammatory amplification after spinal cord injury via IL-18BP up-regulation. European review for medical and pharmacological sciences. 2019;23:1891-8.

5. Xu Z, Yang J, Zheng H, Xie T, Yang Q, Cai J, et al. Long Noncoding RNA PPT2-EGFL8 Regulates Pathological Retinal Neovascularization in PDR by Functioning as a Competing Endogenous RNA. Diabetes. 2023;72:1012-27.

6. Wan W, Liu G, Li X, Liu Y, Wang Y, Pan H, et al. MiR-191-5p alleviates microglial cell injury by targeting Map3k12 (mitogen-activated protein kinase kinase kinase 12) to inhibit the MAPK (mitogen-activated protein kinase) signaling pathway in Alzheimer's disease. Bioengineered. 2021;12:12678-90.

7. Tinsley F, Taicher G, Heiman M. Evaluation of a quantitative magnetic resonance method for mouse whole body composition analysis. Obesity research. 2004;12:150-60.

8. Caccamo A, Branca C, Talboom J, Shaw D, Turner D, Ma L, et al. Reducing Ribosomal Protein S6 Kinase 1 Expression Improves Spatial Memory and Synaptic Plasticity in a Mouse Model of Alzheimer's Disease. The Journal of neuroscience : the official journal of the Society for Neuroscience. 2015;35:14042-56.

9. Wu S, Han L, Lu B, Wang H, Zheng C. MiR-99a inhibits cell proliferation of nasopharyngeal carcinoma by targeting mTOR and serves as a prognostic factor. European review for medical and pharmacological sciences. 2019;23:2053-61.

Source data for figure


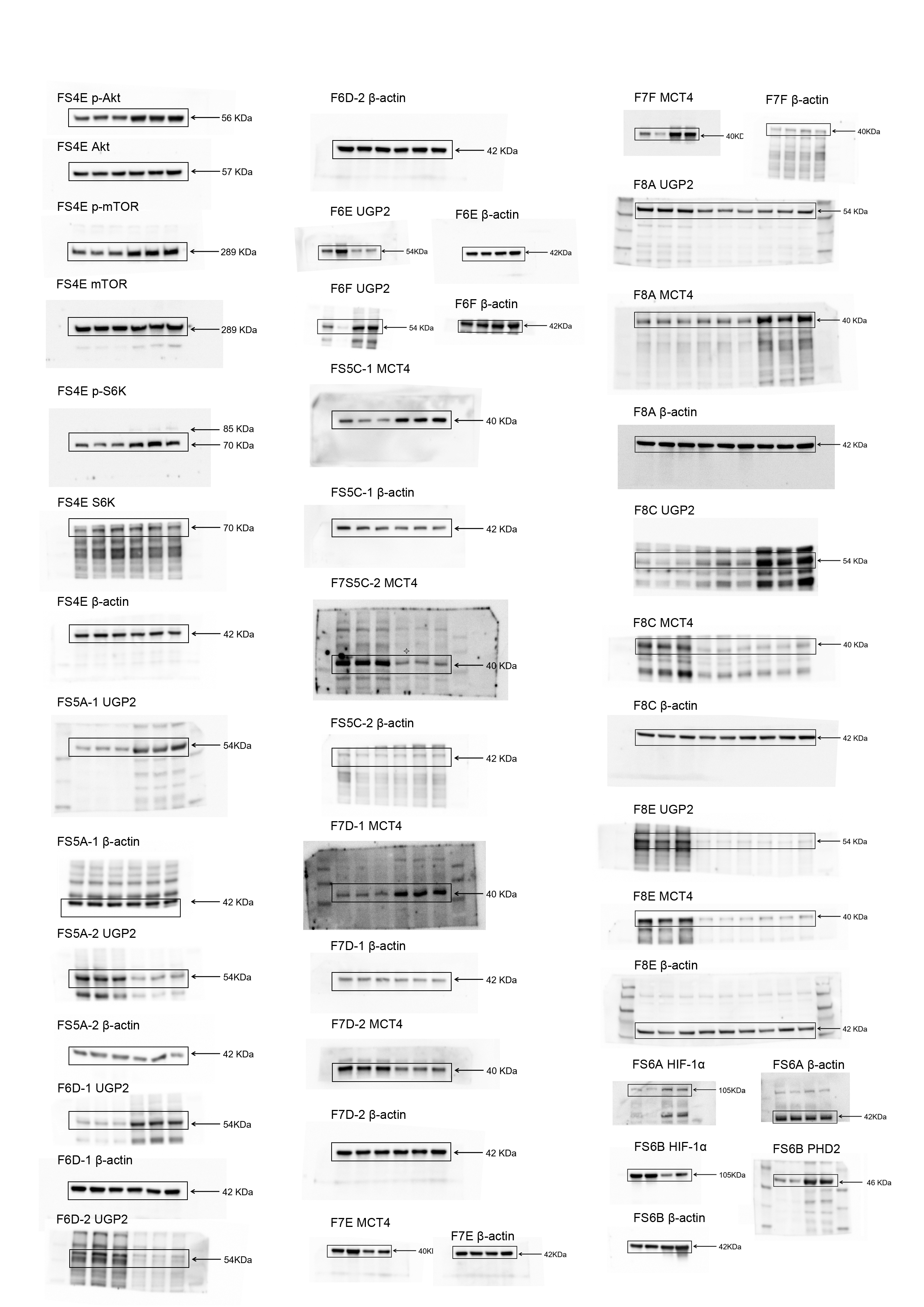

Supplement: Supplementary file 1 — Table S1. The sequences of primers for qPCR analysis. Table S2. Antibodies Information. Figure S1. Skeletal muscle weight in hindlimb suspension mice. (A, B) The weight of GAS and TA of WT mice in control, unload, and unload with reload groups (n = 6). All results are expressed as means ± SD. *p < 0.05, **p < 0.01, ***p < 0.001, by a one‐way ANOVA. Figure S2. MiR‐92b knockout has no evident effect in blood glucose. (A) The qPCR was used to determine miR‐92b‐3p and miR‐92b‐5p in Gas muscle from WT and miR‐92b knockout (M92KO) mice, n = 6. (B) The ratio of heart weight (HW; mg) and tibia length (TL; mm) were shown in panel B (n = 6). (C) Daily food intake were shown in panel C (n = 6). (D) The fasting blood glucose and refed blood glucose in WT or M92KO mice (n = 6). All results are expressed as means ± SD. *p < 0.05, **p < 0.01, ***p < 0.001, by unpaired Student's t test. Figure S3. MiR‐92b overexpression has no evident effect in blood glucose (A) The qPCR was used to determine miR‐92b‐3p and miR‐92b‐5p in Gas muscle from WT and miR‐92b overexpression (M92OE) mice, n = 6. (B) The fasting blood glucose, fasting blood insulin and refed blood glucose in WT or M92OE mice (n = 6). All results are expressed as means ± SD. *p < 0.05, **p < 0.01, ***p < 0.001, by unpaired Student's t test. Figure S4. MiR‐92b knockout improves insulin resistance in diabetic mice. (A) WT or M92KO mice fed with HFD diet and treated with STZ to establish a diabetes model. (B) The qPCR was used to determine miR‐92b‐3p and miR‐92b‐5p in Gas muscle from WT and M92KO diabetic mice, n = 6. (C) The ratio of heart weight (HW; mg) and tibia length (TL; mm) were shown in left panel (n = 6). Daily food intake were shown in right panel (n = 6). (D) The fasting blood glucose, fasting blood insulin and refed blood glucose in diabetic mice (n = 6). (E) Western blot analysis of p‐Akt, Akt, p‐mTOR, mTOR, p‐S6K, S6K, and β‐actin were shown in the panel F, and the quantitative result were shown in the panel M (n [file JCSM-14-2925-s001.docx]
